# Clinical and Translational Landscape of Viral Gene Therapies

**DOI:** 10.3390/cells13221916

**Published:** 2024-11-19

**Authors:** Alexandra Yudaeva, Anastasiya Kostyusheva, Artyom Kachanov, Sergey Brezgin, Natalia Ponomareva, Alessandro Parodi, Vadim S. Pokrovsky, Alexander Lukashev, Vladimir Chulanov, Dmitry Kostyushev

**Affiliations:** 1 Laboratory of Genetic Technologies, Martsinovsky Institute of Medical Parasitology, Tropical and Vector-Borne Diseases, First Moscow State Medical University (Sechenov University), 119991 Moscow, Russia; aleksa.yudaeva@gmail.com (A.Y.); kostyusheva_ap@mail.ru (A.K.); kachanov.av99@gmail.com (A.K.); seegez@mail.ru (S.B.); ponomareva.n.i13@yandex.ru (N.P.); alexander_lukashev@hotmail.com (A.L.); 2 Division of Biotechnology, Sirius University of Science and Technology, 354340 Sochi, Russia; aparodi.sechenovuniversity@gmail.com (A.P.); vadimpokrovsky@yandex.ru (V.S.P.); 3 Department of Pharmaceutical and Toxicological Chemistry, Sechenov First Moscow State Medical University, 119146 Moscow, Russia; 4 Blokhin National Medical Research Center of Oncology, 115478 Moscow, Russia; 5 Department of Biochemistry, People’s Friendship University, 117198 Moscow, Russia; 6 Research Institute for Systems Biology and Medicine, 117246 Moscow, Russia; 7 Department of Infectious Diseases, First Moscow State Medical University (Sechenov University), 119991 Moscow, Russia; vladimir@chulanov.ru; 8 Faculty of Bioengineering and Bioinformatics, Lomonosov Moscow State University, 119234 Moscow, Russia

**Keywords:** adeno-associated viruses, viral vectors, toxicity, oncogenesis, immune response, adverse events

## Abstract

Gene therapies hold significant promise for treating previously incurable diseases. A number of gene therapies have already been approved for clinical use. Currently, gene therapies are mostly limited to the use of adeno-associated viruses and the herpes virus. Viral vectors, particularly those derived from human viruses, play a critical role in this therapeutic approach due to their ability to efficiently deliver genetic material to target cells. Despite their advantages, such as stable gene expression and efficient transduction, viral vectors face numerous limitations that hinder their broad application. These limitations include small cloning capacities, immune and inflammatory responses, and risks of insertional mutagenesis. This review explores the current landscape of viral vectors used in gene therapy, discussing the different types of DNA- and RNA-based viral vectors, their characteristics, limitations, and current medical and potential clinical applications. The review also highlights strategies to overcome existing challenges, including optimizing vector design, improving safety profiles, and enhancing transgene expression both using molecular techniques and nanotechnologies, as well as by approved drug formulations.

## 1. Introduction

Gene therapy is a rapidly advancing field that offers the potential to treat previously incurable diseases by modifying gene expression. In the early 1960s, the classical concept of gene therapy emerged as the delivery of a functional gene copy compensating a defective gene [1]. Since then, the definition of gene therapy has expanded. Gene therapy today includes gene-editing and gene-smodifying technologies (e.g., based on ZFNs, TALENs, or CRISPR-Cas systems), RNA interference (RNAi)-based gene silencing, immunotherapy, stem cell technologies, and vaccine development [2,3,4,5,6]. FDA-approved gene therapies such as Spinraza, Zolgensma, and Elevidys, have played a key role in improving current treatments for neuromuscular disorders [7,8].

There are two different approaches to performing gene therapy: ex vivo and in vivo, depending on how the treatment is administered [9]. The ex vivo approach involves modifying the patient’s own cells by introducing a therapeutic transgene and then injecting the modified cells back into the patient. The in vivo approach refers to directly introducing the therapeutic gene sequence into the bloodstream (systemic administration) or target organs (local administration). Both approaches require efficient gene delivery to target cells, which should result in stable expression of the therapeutic molecules without severe cytotoxicity or inflammatory response. In addition, when delivered in vivo, the transgene must pass through many systemic, extracellular, and intracellular biological barriers and preserve its functionality while reaching the target tissue [10]. Ex vivo gene therapy is employed when it is feasible to achieve the desired therapeutic outcome through in vitro gene transfer of a limited number of autologous cells, followed by their subsequent reimplantation. This ex vivo approach is frequently utilized to treat hematological diseases. The gene of interest is integrated into the genome of autologous hematopoietic stem cells (HSCs) ex vivo and then reimplanted. After implantation, the genetically modified HSCs give rise to blood cells that retain the genetically introduced corrections [10]. Similarly, chimeric antigen receptors are introduced into autologous T cells during the production of CAR-T cell therapy [11]. In contrast, in vivo gene therapy is used when it is necessary to deliver genes directly into specialized cells within the patient’s organism, such as neurons or hepatocytes. In this case, the gene of interest is not integrated but is introduced into the nucleus in an episomal form to avoid insertional mutagenesis [10]. To protect naked nucleic acids from the biological environment and improve intracellular targeting, so-called “delivery vectors” are used [12]. These vectors can be categorized as either viral or non-viral based on their properties [13].

The majority of viral vectors are derived from human viruses that have been modified to be harmless by removing essential genes for replication. Compared to non-viral vectors, these viral delivery systems offer a significant advantage due to their ability to efficiently cross different biological barriers, infect cells, and establish stable gene expression within target tissues, but their main drawback is their immunogenicity [10]. Over 70% of clinical trials for gene therapies are based on viral vectors [14]. Non-viral vectors, in contrast, are generally considered to be less toxic and immunogenic; however, they are less effective at traversing biological barriers, such as the endosomal barrier [15].

**Figure 1:**
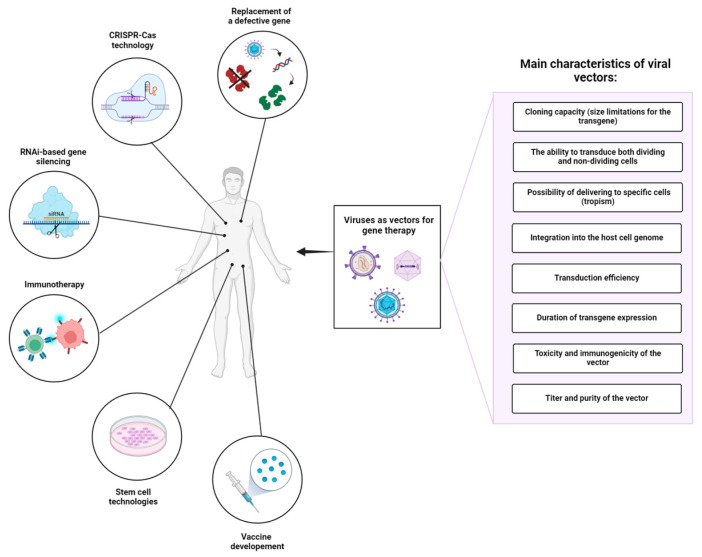
Application of viral vectors in gene therapy and important factors influencing the choice of viral vector platform. In addition to gene replacement therapy, viral vectors are used as platforms for gene modifying technologies (e.g., CRISPR-Cas systems), RNA interference (RNAi)-based gene silencing, immunotherapy, stem cell technologies, and vaccine development. In summary, the choice of viral vector platform in gene therapy is influenced by a combination of biological properties, safety considerations, and practical factors related to manufacturing and regulatory approval. Ongoing research and technological advancements continue to expand the repertoire of available viral vectors, offering new possibilities for treating a wide range of genetic and acquired diseases.

Viruses display a variety of types and species differing in genome size, type of genetic material, morphology of the viral capsid, tropism, etc. [16]. Derived from numerous types of viruses, viral delivery systems can be classified according to the type of genetic material in DNA-based and RNA-based vectors. When selecting a viral vector for a particular gene therapy application, it is crucial to consider the following factors (Figure 1): (1) cloning capacity (size limitations for the transgene), (2) the ability to transduce both dividing and non-dividing cells, (3) tropism of viruses to specific cells/tissues, (4) integration into the host cell genome, (5) transduction efficiency, (6) duration of transgene expression, (7) toxicity and immunogenicity of the vector, and (8) titer and purity of the vector (Table 1). An ideal viral vector should have a high cloning capacity, ensure exclusive transduction of target cells, and establish stable, lifelong transgene expression. In addition, such a viral vector must meet high safety standards: be safe for the patient and handling personnel, lack potential risks of tumorigenesis, and cytotoxic and immune-associated side effects. The production of such vectors should be cost-effective, reproducible, and easily scalable, providing high titers of infective viral particles, and lacking contaminants and any other impurities. Despite the variety of viruses that can serve as a basis for developing viral delivery systems, modern viral vectors do not come even close to these standards.

In addition, oncolytic viruses have emerged as effective cancer immunotherapies over the past few decades [17]. Oncolytic viruses induce an antitumor reaction by infecting and destroying tumor cells, while also triggering the activation or restoration of the body’s natural antitumor defense mechanisms. Oncolytic viruses are engineered to selectively replicate in tumor tissues and lyse cancer cells, leaving healthy non-tumor cells unaffected. In addition, they can also be engineered to carry coding sequences for co-stimulatory molecules, cytokines, and antibodies that can additionally inhibit the immunosuppressive tumor microenvironment, mounting efficient antitumor responses [18].

Numerous viral vectors have been authorized for medical use as reliable and secure tools for gene therapy. Despite remarkable success in treating previously incurable diseases, these vectors still elicit severe immediate and long-term toxicity, can pose significant health risks and rarely cure a disease completely [19,20]. This review addresses the inherent problems of viral vectors hindering their safety and efficacy in different applications of gene therapies, including cancer immunotherapy.

## 2. DNA-Based Viral Vectors for Gene Therapy

### Adenoviral Vector

Adenoviruses are a group of viruses that belong to the Adenoviridae family [21]. They are non-enveloped and have an icosahedral capsid of 90–100 nm in diameter. The adenoviral genome is double-stranded DNA with its length varying between 26 and 45 kbp [22,23]. Human adenoviruses are divided into seven species or groups (A–G). Across the species, they are further divided into types based on identification either with serum neutralization (serotypes 1–52) or through genotyping (genotypes 53–103) [23,24]. Adenoviral vector (AdV) of serotype 5 is the most frequently studied adenovirus in gene therapies [23]. Human adenovirus serotype 5 (HAdV-5) has a 36 kbp long genome that accommodates 38 viral genes subdivided into 17 transcriptional units: early units (E1–E4), intermediate units, and late units (L1–L5). E1 unit encodes proteins involved in viral DNA replication initiation, E2 and E4 units encode proteins responsible for transcription, and E3-encoded proteins are responsible for host responses for adenoviral infection evasion. Intermediate units encode proteins IX and IVa2. The late transcriptional units encode viral capsid proteins [25].

Adenovirus was first isolated in 1953. However, it was only after nearly 40 years that the first in vivo gene transfer with an adenoviral vector was performed [26,27]. In 1991, Melissa Rosenfeld and colleagues were able to deliver recombinant the α1-Antitrypsin gene to the murine lung epithelium in vivo. The vector genome lacked part of the E1a region, which was responsible for viral replication, and part of the E3 region, responsible for genome encapsidation [27]. Later, the type of replication-dependent AdV with E1 and/or E3 deletions would be termed first-generation AdV (FgAd) [28]. In 1993 and 1994, adenovirus-mediated gene transfer was successfully performed in humans [29,30]. However, studies of FgAd have exposed several significant drawbacks of this vector. Transgene expression duration was limited, and re-administration was necessary. Re-administration of AdV resulted in progressively lower levels of transgene expression. FgAd could also cause immune-related adverse events [31,32]. In order to mitigate these disadvantages, in 1994, the second generation of AdV was created through the E2a gene mutation [33]. Second-generation AdV had a higher cloning capacity of up to 14 kbp and lower immunogenicity in comparison to first-generation AdV [34]. In order to even further reduce AdV immunogenicity, while simultaneously enhancing cloning capacity, gutless AdV was developed in 1997 [35]. Gutless AdV is devoid of all viral DNA, providing a cloning capacity of up to 36 kbp and prolonging transgene expression [34].

Despite these advancements, the unfavorable immunogenic properties of AdV as an in vivo gene therapy vector resulted in the first death of a patient directly related to the gene therapy [36]. Jesse Gelsinger died from acute respiratory distress syndrome due to systemic inflammation, caused by the administration of 6 × 10^11^ vg/kg (3.8 × 10^13^ vg in total [37]) of the E1- and E4-deleted adenovirus vector, containing the human ornithine transcarbamylase transgene [38]. In spite of high immunogenicity, the field of application for the adenoviral vector is now somewhat limited to oncolytic therapy and vaccine studies, where its immunogenic properties are considered to be beneficial [39]. Moreover, adenovirus vectors are currently utilized at significantly lower doses. For instance, a COVID-19 vaccine has a total dose of only 1 × 10^11^ viral genomes (vg), while AZD1222 has a total dose of 5 × 10^10^ vg, which are respectively 380 and 760 times lower than the dose received by Jesse Gelsinger [40,41]. Oncolytic viruses are administered at higher doses than vaccines; however, these doses remain several times lower than 3.8 × 10^13^ vg [42]. The use of lower doses in conjunction with local administration methods, such as intramuscular or intratumoral injection, reduces the risk of an acute systemic inflammatory response.

## 3. Immune Response

The immune response to AdV is a significant barrier, hindering its implementation as a gene replacement therapy vector. The immune response to AdV is recognized as a primary factor contributing to several notable limitations associated with their use. These limitations include the rapid clearance of transgene expression, immune-mediated toxicity, and the development of neutralizing antibodies. As a result, these factors significantly impede the potential for vector re-administration. In 1994, during their investigation of FgAd vector-mediated gene transfer into hepatocytes, Yang and colleagues observed that more than 80% of hepatocytes in immunocompetent mice were lacZ-positive on day 2 after the infection. However, lacZ expression was undetectable at day 21. Immunocompetent mice also exhibited significant liver pathology. In contrast, transgene expression in athymic mice was sustained at consistent levels for up to 60 days post-infection and exhibited no liver pathology. Both of these effects were considered to be due to the cellular immune response to the leaky expression of viral E2a and hexon proteins [31]. The rapid decline in transgene expression levels due to cellular immune response was also documented in several other studies in different tissues, such as lung, brain, and muscle tissue [31,43,44,45,46]. Importantly, all of the mentioned studies utilized FgAd without E3 deletion. A functional E3 gene allows the adenovirus vector to suppress the immune response [47].

In response to the observed toxicity associated with the cellular immune response to the first-generation FgAd adenoviral vector, a second-generation adenovirus vector was engineered, incorporating deletions in both the E1 and E2a regions, in order to reduce leaky viral gene expression [48]. Transgene expression was undetectable at day 21 following transduction with an E1-deleted adenoviral vector. In contrast, transduction with a second-generation adenoviral vector resulted in detectable lacZ expression even at day 70, accompanied by a reduced inflammatory response in murine liver models [49]. Similar findings were observed in murine lung studies [33]. Additionally, a variant of the second-generation adenoviral vector featuring deletions in both the E1 and E4 regions demonstrated enhanced performance compared to FgAd vectors. Specifically, over 50% of murine hepatocytes exhibited lacZ positivity 60 days post-transduction with the E1/E4-deleted adenoviral vector, whereas less than 10% of hepatocytes were positive following transduction with the FgAd vector [50]. In summary, the evidence suggests that leaky viral gene expression was indeed responsible for the rapid decline in transgene expression. However, a study conducted in non-human primates demonstrated that psoralen-inactivated, E1-deleted viral particles, which are incapable of gene expression, still elicited an innate immune response, as evidenced by elevated systemic levels of IL-6. Notably, these levels were even higher than those observed following functional FgAd transduction. Furthermore, both inactive and active FgAd vectors induced splenic histopathological abnormalities. These findings indicate that the immune response to adenoviral vectors is not solely attributable to late gene expression but may also be related to the innate immune response to adenoviral vectors, independent of viral gene expression [51].

In order to further reduce the possibility of immune-related adverse events, third-generation adenovirus vectors were developed. The third generation of adenovirus vectors, also known as gutless (GLAd) or helper-dependent, is an adenovirus vector devoid of nearly all viral genomes. This vector was reported to exhibit prolonged transgene expression, lower tissue infiltration by macrophages, and little to no tissue damage [52]. Additionally, hepatic aminotransferase levels were similar to that of the control in GLAd-transduced mice, whereas FgAd transduction induced severe liver toxicity. Transgene expression was sustained for a minimum of 60 days in mice transduced with the GLAd vector, whereas it exhibited a rapid decline by week 2 in mice treated with the Fg-Ad vector [53]. Similar results were reproduced in several other trials [54,55]. However, despite the enhanced persistence of transgene expression observed with the GLAd vector, the innate immune response elicited by GLAd is comparable to that induced by the FgAd5 vector [56]. The magnitude of this immune response was underscored in a study conducted on non-human primates by Brunetti-Pierri, which further corroborated the findings from the aforementioned study involving psoralen-inactivated adenoviral particles [51,57]. In this study, the animal receiving the lower dose exhibited transient acute toxicity and was euthanized 96 h post-injection. Necropsy revealed that approximately 50% of the liver tissue was lacZ positive, with no gross abnormalities noted. In contrast, the animal administered the higher dose experienced severe acute toxicity and was euthanized 8.5 h post-injection due to its deteriorating condition. Necropsy findings indicated 100% lacZ positivity in the liver, along with partial lung congestion, intestinal hemorrhage, and splenic abnormalities. Both animals displayed signs of hepatic toxicity, characterized by elevated hepatic aminotransferase levels within hours of injection. These events were determined not to be associated with lacZ due to their early onset [57].

In a separate study, transduction of mouse liver using FgAd and HDAd demonstrated similar immune response patterns for both vectors. In contrast, adeno-associated viral vector (AAV), currently the predominant platform for gene replacement therapy, elicited a significantly less pronounced immune response [58].

Thus, GLAd achieved a lower adaptive immune response and prolonged transgene expression; however, it retained a pronounced innate immune reaction similar to that observed with FgAd.

In addition to the cellular and innate immune responses, the humoral immune response represents a significant obstacle to the effective application of AdV in gene therapy. Neutralizing antibodies (NAbs) bind viral capsid and significantly reduce transduction efficiency [59,60]. Pre-existing neutralizing antibodies are highly prevalent; for example, neutralizing antibodies against hAd5 were found in nearly 50% of patients with neuromuscular disorders living in Germany [61]. Seroprevalence can vary in other regions and populations [62]. In a study conducted by Mast et al., it was found that only 14.8% of participants from various regions worldwide lacked NAbs against Ad5 [63]. NAbs are known to be primarily directed against the hexon protein of AdV capsid [64]. Thus, the potential for vector neutralization by NAbs appears to be independent of AdV vector generation. The presence of NAbs can be effectively bypassed through the selection of rare, non-human, or chimeric AdV serotypes [65,66,67,68]. Nevertheless, subsequent vector re-administration may lead to diminished transduction efficiency [69]. Furthermore, coating HAdVs with polyethylene glycol (PEG) facilitates the evasion of neutralizing antibodies and reduces uptake by macrophages and Kupffer cells [70]. Similarly, AdV modified with PEGylated oligopeptide-conjugated poly(β-amino esters) demonstrated reduced toxicity, evaded NAb neutralization, and elicited significantly lower levels of NAb production [71].

### 3.1. Adeno-Associated Viral Vectors

The adeno-associated viral vector (AAV) is a small (20–25 nm in diameter) non-pathogenic parvovirus with a single-stranded DNA genome of approximately 4.7 kb [72]. The AAV genome contains two major open reading frames flanked by two inverted terminal repeats (ITRs) 145 nucleotides long. Structural regions of inverted terminal repeats (ITRs) play a crucial role in various stages of the viral life cycle, including replication, encapsidation, concatenation of the episomal viral genome, and integration into the host genome [73]. Transcription of the first ORF, the Cap ORF, results in the expression of three viral capsid proteins VP1, VP2, and VP3, which form a 60-mer icosahedral capsid. The Cap ORF also encodes membrane-associated accessory protein (MAAP) and the assembly activating protein (AAP), involved in the capsid assembly of some AAV serotypes [74,75]. The second ORF encodes four AAV replication (rep) genes (Rep40, Rep52, Rep68, and Rep 78), which mediate viral replication, transcription regulation, genome integration, and virion assembly [76]. As a dependent parvovirus, AAV requires additional adenovirus helper genes E1, E2a, E4, and VA RNA to achieve productive infection. To produce recombinant AAVs, the gene of interest (GOI) is placed between the ITRs, and the rep and cap genes are removed. These components, along with viral helper genes, are co-delivered into cells during AAV production [77].

AAV has at least 13 natural serotypes and over 100 variants, some of which were artificially created using molecular engineering techniques. Each serotype exhibits its own tissue tropism, defining the AAV biodistribution profile upon systemic administration. Tissue tropism depends on the specific interactions between structural elements of the AAV capsid with primary cell surface glycoprotein receptors (for example, heparan sulfate for AAV2, AAV3, AAV6, and AAV13) and secondary receptors or coreceptors that facilitate their internalization (for example, fibroblast growth factor receptor 1 (FGFR1), CD9, avβ5 integrin, and α5β1 integrin for AAV2) [78,79]. The combination of these receptors is believed to be a key factor determining the tissue tropism of AAV serotypes. AAV efficiently transduces both dividing and nondividing cells, establishing stable transgene expression and relatively low (compared to adenoviruses) immunogenicity.

Due to these features, AAV vectors are currently the leading delivery system for in vivo gene therapy with five AAV-based drugs approved by the FDA to date. These include Luxturna for the treatment of retinal dystrophy (Leber congenital amaurosis), Zolgensma (spinal muscular atrophy), Hemgenix (hemophilia B), Elevidys (Duchenne muscular dystrophy), and Roctavian (hemophilia A). Over 100 AAV-based drugs are currently in clinical trials focused on hereditary eye diseases, lysosomal storage diseases, blood disorders, central nervous system conditions, and neuromuscular disorders [80]. Despite the extensive range of AAV drugs available for treating various diseases, several limitations hinder the widespread utilization of these vectors.

#### 3.1.1. Small Cloning Capacity

AAV has a compact genome, which limits the size of the insert to 5 kb between ITR sequences. Exceeding the packaging capacity of AAV leads to the formation of viral particles with a partial genome [81]. This limits the use of AAV to deliver large inserts, such as Cas proteins, coagulation factor 8, dystrophin, dysferlin, myosin VII, Cystic Fibrosis Transmembrane Conductance Regulator (CFTR), ATP Binding Cassette Subfamily A Member 4 (ABCA4), etc. For example, the length of SpCas9 cDNA is ~4.1 kb, and it cannot be placed in a single vector. Moreover, single-guide RNA (sgRNA) is necessary for the recognition of the target sequence by Cas9 protein. Due to size restrictions of AAVs, sgRNAs are typically delivered separately from Cas9 proteins. Smaller, evolved versions of Cas9 protein or orthologous proteins from different species, such as *Streptococcus aureus* (SaCas9; 3.2 kb), *Streptococcus thermophilus* (St1 Cas9), and *Campylobacter jejuni* (CjCas9; 2.95 kb) can be used to overcome transgene size limitations [82,83]. Alternatively, the transgene sequence can be optimized by removing domains that are not essential for its biological activity. This strategy was implemented for Factor VIII, an essential plasma clotting protein, whose deficiency is responsible for hemophilia A. Gene therapies based on AAV utilize a shortened, but fully functional version of FVIII factor, FVIII-SQ. Examples of this approach are minidystrophin for the treatment of muscular dystrophy, and B-domain-deleted coagulation factor FVIII (BDD-FVIII) for the treatment of hemophilia A [84,85]. The highly glycosylated B domain is involved in the intracellular processing by providing sites for cleavage. Although complete deletion of this domain does not affect the coagulation function of FVIII, deletion of different regions of the B domain has different effects on protein expression, intracellular transport, secretion, and stability [86]. Thus, the identification of functionally important protein domains and their regions using structural biology techniques provides the opportunity to create AAV drugs with reduced variants of large genes for the treatment of other diseases in the future.

**Figure 2:**
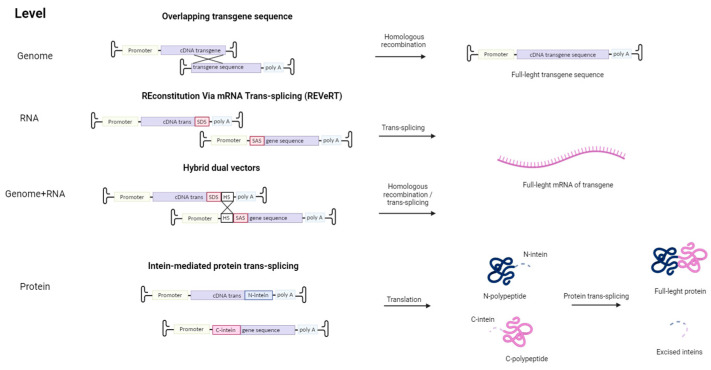
Strategies to overcome the limited cloning capacity of AAV by using multiple viral vectors. First, to overcome the limited cloning capacity of AAV, several AAV vectors carrying two parts of the transgene with overlapping sequences can be used. Subsequently, the full-length transgene is formed as a result of homologous recombination in the cell. The REVeRT technology is an mRNA-based approach. The full-length version of the transgene is reconstituted in the cell through trans-splicing, which is facilitated by the splice donor site (SDS) and splice acceptor site (SAS) in the viral vector. The combination of the REVeRT technology approach with an overlapping sequence (HS—homologous site) for homologous recombination is called hybrid dual vectors. Also, restoration of a split transgene is possible at the protein level through intein-mediated trans-splicing.

Other strategies were implemented to overcome the limited cloning capacity of AAVs (Figure 2). For example, the transgene containing overlapping sequences is split into several AAV vectors. Inside the cell, the transgenes are assembled into a single coding gene via homologous recombination. Alternatively, such recombination may occur at the protein level with the use of inteins—protein sequences embedded between divided parts of the large protein. These split protein sequences are then effectively trans-spliced producing a single, functional protein [87,88]. However, this approach requires proper folding of both split proteins, and intein proteins are potentially immunogenic. This issue can be avoided by using an mRNA trans-splicing approach to deliver large transgenes using AAV. The first vector contains a splice donor site (SDS), and the second vector—a splice acceptor site (SAS), so in the cell full-length sequence is restored via mRNA trans-splicing (REVeRT). The REVeRT technology showed similar efficacy compared to the split-intein AAV cassette in a mouse model for subretinal delivery of dual AAV8 vectors encoding Cas9-VPR and three sgRNAs [89]. AAV genome-level reconstruction approach and a trans-splicing approach were combined together in a technology called hybrid dual vectors [90]. Thus, employing two vectors enables an expansion in the size of the transferred transgene to 9 kb, while for triple AAV vectors, the size can be enlarged up to 14 kb.

The major drawback of this split strategy is that restoration of a full-length functional protein requires simultaneous transduction of the same cell with at least two viral vectors in the same or similar proportions. Indeed, Colella et al. showed that expression efficacy of dual vectors is 2–3 times lower than that of single vectors [91].

Currently, about 50% of drugs undergoing clinical trials are based on the use of strong constitutive promoters, such as CBA (chicken beta-actin), CMV (cytomegalovirus), or CAG (a synthetic promoter consisting of a CMV enhancer, CBA promoter, and a rabbit beta-globin splice acceptor). However, the use of ubiquitous and tissue-specific promoters varies depending on the therapeutic area. Constitutive promoters predominate in the treatment of diseases of the central nervous system, while for the treatment of blood diseases, 95% of drugs use tissue-specific promoters [80]. The ratio is primarily influenced by drug delivery methods and is closely tied to minimizing adverse effects associated with systemic drug administration. While constitutive promoters provide robust and sustained transgene expression, they can also induce cytotoxic responses due to overexpression, off-target gene expression, or immune reactions triggered by transgene presentation in antigen-presenting cells. In addition, the size of such promoters ranges from ~500 (CMV) to 1000 bp. (CAG) and more, which further reduces the capacity of AAV for transgene cloning. The strategy for searching for optimal promoter variants consists of either testing various truncated promoter sequences of natural genes, or assembling hybrid promoters using elements from various known enhancers and promoters [79]. In order to find the optimal promoter for gene therapy of retinal degeneration, the main promoter of human rhodopsin kinase, which is tissue-specific for photoreceptors, was found [92]. The development of so-called micropromoters, MP-84 and MP-135, whose DNA sequence is derived from the human insulin and glucagon promoter regions, respectively, has also been reported. These micropromoters are only 84 (MP-84) and 135 bp (MP-135). In addition, they demonstrate high transgene expression in vivo, comparable to such a strong promoter as CAG, in skeletal muscle of a mouse model and in human hepatocytes of liver-humanized mice [93]. In addition, computational approaches allow the generation of libraries of fully synthetic promoters by identifying transcription factor binding sites and other cis-acting sequences using microarrays and genome-wide functional assays, which are then utilized as components in synthetic promoters [94]. Thus, the muscle-specific SPc5-12 promoter was developed and used to express canine microdystrophin in an in vivo experiment. This transgene, delivered systemically using the rAAV2/8 vector, showed high levels of microdystrophin expression in canine limb musculature for at least 26 months without toxicity or adverse immune reactions [95]. A library of over 1000 promoter variants was created for the search. These variants consisted of the minimal promoter of the chicken alpha-skeletal actin gene and shared binding sites for myogenic transcription factors (SRE, MEF2, MEF1, and TEF1) that were derived from strong muscle promoters and randomly ligated to each other in forward and reverse orientation.

#### 3.1.2. Loss of Episomes in Replicating Cells

After AAV vectors enter the host cell, their single-stranded genome is converted into double-stranded circular episomal forms, which persist for a long period and determine the stable expression of the transgene in postmitotic cells [96,97]. Studies in large animal models, including dogs and non-human primates, have demonstrated sustained expression and physiological activity of the factor IX transgene for at least five years following a single systemic or intramuscular AAV vector administration [98,99]. However, it is still unclear whether the vector episomes will persist and provide stable expression throughout life. In addition, fast cell proliferation contributes to the dilution and loss of AAV episomes [100]. Thus, rapidly proliferating cells such as hematopoietic stem and progenitor cells are rarely considered an appropriate target for AAV-based gene therapy [101]. In the liver, in addition to hepatocyte division due to physiological growth, the persistence of AAV may also be negatively affected by fibrosis and cirrhosis of the parenchyma [102].

To counteract the dilution effect and enhance gene therapy efficacy in rapidly dividing cells and neonatal liver, researchers are actively developing various strategies. They usually aim at integrating the viral genome into the genetic material of the host cells [103,104,105]. The most universal method is the use of the CRISPR-Cas system, the endonuclease of which induces a double-strand break in the target region of the host genome due to specific sequence recognition through guide RNA. Consequently, double-strand break repair mechanisms are activated, including homologous recombination, which utilizes the transgene provided with the CRISPR-Cas system as a template [101,106]. The effectiveness of intravenous co-delivery of the CRISPR-Cas system with the transgene using dual AAV vectors was demonstrated in a hemophilia B mouse model, as well as in an ornithine transcarbamylase-deficient mouse model [107,108,109]. In addition, this system can be used to suppress gene expression of genes carrying pathological mutations through another mechanism of double-strand break repair, non-homologous end joining (NHEJ) [101]. Technologies aimed at integrating AAV into the genome may be promising approaches for maintaining long-term transgene expression in dividing cells with a low risk of insertional mutagenesis through controlled integration. Nevertheless, it is important to note that CRISPR-Cas technology has several significant drawbacks that restrict its use in therapy and clinical settings. A major problem of CRISPR-Cas is the high frequency of mutations induced by off-target activity at sites different from the intended target site [110]. In addition, as mentioned above, the use of dual AAV vectors is less effective compared to delivery using a single AAV vector.

#### 3.1.3. Immune Response and Immune-Mediated Toxicity

**Figure 3:**
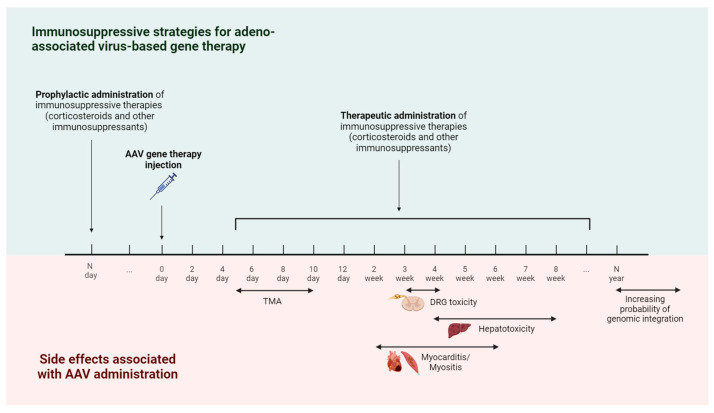
Timeline of immune-mediated adverse events and immunosuppressive strategies for AAV-based gene therapy. The earliest immune-mediated side effect caused by AAV injection is thrombotic microangiopathy (TMA), which occurs 5–10 days after treatment. Starting from the second week, there is a risk of developing side effects such as hepatotoxicity, myocarditis, myositis, and dorsal root ganglia (DRG) toxicity. In order to reduce the risk of developing immune-mediated side effects, immunosuppressive therapy is used. The administration of immunosuppressive drugs is possible in two modes—prophylactic administration (carried out before AAV gene therapy) and therapeutic administration (carried out in response to the appearance of immune-mediated side effects).

Administration of AAV vectors elicits a less potent immune response compared to other viral vectors like adenovirus. However, the immune reaction to AAV may contribute to several adverse effects observed in clinical trials and continues to pose a significant obstacle to translating this technology (Figure 3). Since most of the AAV viral genome is removed or optimized to lack immunogenic CG nucleotides, the immune response is mostly directed to antigens of the viral capsid and transgene cassette [12]. In addition, the activation of the immune response is also influenced by the nature of the transgene, the administered dose, and the route of drug administration [111]. Patients who received AAV therapy via intravenous infusion are at higher risk due to interactions of the vector with immune cells and plasma proteins, including antibodies and the complement system.

##### Innate Immune Response

Upon the entry of AAV vectors into the body, the innate immune response is quickly triggered by key elements like pattern recognition receptors and the complement system. Thus, among pattern recognition receptors, the main role is played by endosomal TLR9, which, in particular, binds to unmethylated CpG dinucleotides of vector DNA [112,113]. Also, innate immunity is triggered by AAV genome recognition by cytosolic DNA sensors, such as cyclic GMP-AMP synthase (cGAS) [114]. Binding of AAV vectors by these receptors stimulates pro-inflammatory signaling cascades aimed at recruiting and activating antigen-presenting cells, T cells, and B cells, which in turn will contribute to the development of an adaptive immune response [115]. Optimization of AAV genomes by deleting or substituting CpG nucleotides attenuated innate immune recognition of AAV genomes [116].

An important player in the innate immune response to AAV is the complement system, comprising a group of plasma proteins. Three pathways can activate complement, including the classical, lectin, and alternative pathways, ultimately forming the membrane attack complex (MAC). Activated complement proteins stimulate the uptake of viral particles by macrophages, opsonizing their capsids, triggering the activation and chemotaxis of immune cells, and their subsequent release of pro-inflammatory cytokines, such as IL-8 and IL-1β. In addition, the complement system can trigger blood clotting pathways and platelet activation, which can lead to microvascular damage and blood clots [117,118]. In turn, this can lead to the development of thrombotic microangiopathy (TMA).

TMA is one of the most common severe complications, which mainly occurs with systemic administration of high doses of AAV usually 5–10 days after treatment [119]. This adverse reaction is believed to arise from heightened stimulation of the complement system via both the classical pathway (triggered by anti-capsid antibodies) and the alternative pathway (initiated through direct interaction of the viral capsid with the C3 protein) [120]. TMA is associated with either direct increased platelet aggregation, activation of the coagulation cascade, or activation or damage to endothelial cells. All these mechanisms lead to thrombosis, thrombocytopenia, and hemolytic anemia [121,122]. The precise interplay between AAV and the host organism that results in TMA, and whether it is related to complement activation or endothelial transduction by AAV, remains undefined and is discussed in detail in a recent mini-review by Schwatzer et al. [122]. As of October 2024, at least 13 cases of TMA in patients undergoing viral-vector-based gene therapies have been reported. Eight of these cases occurred following onasemnogene abeparvovec treatment for spinal muscular atrophy, with affected patients ranging in age from 5 months to 4 years [121]. One case resulted in the death of a 6-month-old patient on day 57 after gene therapy administration [123]. In the aforementioned cases, the onset of TMA occurred 5 to 8 days post-treatment [121]. Additionally, four cases of TMA associated with complement activation were reported among a total of 25 patients who received two different AAV-based therapies for Duchenne muscular dystrophy (DMD) in separate clinical trials [124,125]. No further demographic information regarding the affected patients or the timing of onset is available [124,125]. In a case involving high-dose AAV9 (1.1 × 10^14^ vg/kg) administered for Danon disease, one of two pediatric patients aged 11 and 12 years developed TMA with acute renal failure [121,126].

Eculizumab was used to prevent complement hyperactivation in clinical trials of AAV gene therapy in SMA and DMD [127]. This drug is a monoclonal antibody against complement protein C5, which prevents the formation of the pro-inflammatory peptide C5b and cytotoxic MAC [128]. Although eculizumab is recommended for the treatment of TMA caused by AAV gene therapy, the effect of this therapy on clinical outcomes has been inconsistent, suggesting that further research is needed. As this complication has the potential to be life-threatening, it is advisable to assess risk factors for TMA (such as infections, complement inhibitor antibodies, and genetic predispositions) and evaluate the activity of the complement system before starting the treatment. This will allow for a subsequent assessment of post-treatment kinetics [123].

##### Adaptive Immune Response

AAV vector administration can lead to serious complications of TMA as a result of the innate response, but the majority of adverse effects are attributed to the adaptive immune response [129]. The adaptive immune response to AAV can be divided into two main components: humoral immunity, which consists of the production of specific antibodies against the vector serotype, and cell-mediated immunity, mediated by cytotoxic CD8+ T-lymphocytes [130].

##### Neutralizing Antibodies and Cell-Mediated Immune Response

Neutralizing antibodies generated in response to AAV administration poses a serious challenge for clinical use, since such anti-capsid antibodies are capable of neutralizing viral particles even at low titers, thereby reducing the efficiency of cell transduction [131,132]. This limits the repeated administration of drugs based on an adeno-associated vector of the same serotype. However, in the case of neonates and children, and patients with degenerative disorders, repeated administration of the AAV vector may be necessary due to the dilution or the loss of the vector genomes [133]. In addition, pre-existing neutralizing antibodies to AAV that were generated through natural exposure to wild-type AAV viruses are widespread in the human population [134]. Their prevalence in human serum varies from 30% to 80% and is highly dependent on geography and AAV serotype. Anti-AAV2 and anti-AAV1 neutralizing antibodies prevail among antibodies to other serotypes [135,136].

Currently, patients with neutralizing antibodies above a specific level in their bloodstream are frequently not allowed to participate in clinical trials. This criterion is used to avoid immune-mediated side effects and neutralization of the viral vectors when administered systemically, primarily in the treatment of blood diseases and neuromuscular diseases [80]. Unfortunately, due to the high prevalence of immunological memory for AAV capsid antigens in most people, this limitation excludes a significant proportion of patients from participation in clinical trials. Plasmapheresis may be used to reduce circulating antibody levels; however, this method is not able to completely eliminate AAV-neutralizing antibodies and may require several cycles of plasmapheresis. In a 2011 trial, ten patients underwent 2 to 5 cycles of plasmapheresis to eliminate NAbs. Plasmapheresis significantly reduced NAb quantities; however, non-detectable or low (<1:5) antibody levels were only achieved in patients with low initial NAb levels (<1:20). Additionally, a rebound effect was observed, as antibody levels increased again following successful plasmapheresis due to renewed antibody production [137]. Despite this, two separate studies on rhesus macaques demonstrated that plasmapheresis achieved similar transgene expression levels in both seropositive and seronegative animals, and also facilitated safer AAV redosing [138,139]. However, there are currently no available results from clinical trials utilizing plasmapheresis in conjunction with gene therapy.

In some cases, the binding of the viral particles by neutralizing antibodies can be avoided by changing the systemic route of administration to local. Thus, direct delivery of AAV to the central nervous system is less sensitive to pre-existing antibodies, due to their lower concentration in cerebrospinal fluid compared to the bloodstream [140]. However, in this case, the biodistribution of the AAV vector and transduction efficiency may be less efficient. A commonly employed approach involves the utilization of B cell-depleting monoclonal antibodies, such as rituximab and belimumab, and the combination of rituximab with the mTOR inhibitor rapamycin, or with rapamycin and corticosteroids, in order to prevent nAb formation and enable re-administration [141,142,143]. For example, in a case study, two patients were treated with rAAV-based therapy alongside an immunosuppressive regimen consisting of prednisolone, rapamycin, and rituximab [142]. Similar results were observed in another study, where patients not receiving an immunosuppressive regimen demonstrated at least a 155-fold increase in anti-AAV titers. In contrast, the patient receiving the combination of rituximab, sirolimus, and methylprednisolone did not exhibit any increase in anti-AAV antibody titers above baseline; moreover, the T-cell response against the capsid remained unchanged [141]. Another promising strategy is tolerogenic nanoparticles containing rapamycin (ImmTOR), which, when co-administered with AAV vectors, provide dose-dependent and long-term suppression of humoral and T-cell responses against AAV. In a study on a murine model, ImmTOR was successful at preventing Nab formation. While Nabs were detectable in the serum of mice treated solely with rAAV, no NAbs were detected in the serum of mice that received rAAV combined with ImmTOR [144]. Modification of antigenic epitopes on the AAV capsid has been also investigated to reduce the adaptive immune response to viral vectors. Currently, there are several main methods for generating alternative AAV serotypes, including the search for new natural variants, rational design, directed evolution, and in silico approaches. You can learn more about strategies for reducing innate and adaptive immune responses to adeno-associated vectors in the following reviews [79,127,133].

#### 3.1.4. Hepatotoxicity

The liver is typically the primary destination for all AAVs, independently from their serotype and tissue affinity [111]. This increases the potential for liver damage when these vectors are delivered systemically. Hepatotoxicity is a frequent complication observed in the majority of clinical research, typically associated with increased systemic levels of liver enzymes such as ALT and AST. In the first clinical trials of gene therapy for hemophilia, liver damage was documented when AAV2 was employed to deliver human blood clotting factor FIX [145]. Signs of hepatotoxicity were also reported in about a third of patients treated with onasemnogene abeparvovec for SMA [146]. Two individuals in this clinical research experienced significant liver damage characterized by hepatocyte degeneration and inflammatory infiltration [147]. After administration of an AAV drug for the treatment of X-linked myotubular myopathy, four patients died due to severe hepatotoxicity. However, all four patients had liver pathologies before receiving the treatment [146,148].

In most cases, hepatotoxicity was observed 4–8 weeks after administration of therapy and was manifested in an increase in the level of liver transaminases and a decrease in transgene expression. Elevated levels of liver transaminases, along with the presence of AAV-specific CD8+ T cells in the majority of patients, suggest that hepatotoxicity caused by adeno-associated vector gene therapy may arise from an adaptive immune response to AAV capsids mediated by T cells [134,149]. In vitro studies have shown that the elimination of hepatocytes by capsid-specific CD8 + T cells occurs through the cross-presentation of capsid proteins to MHC I [150].

Elevated levels of liver enzymes and bilirubin, along with hepatocyte necrosis, have also been documented in preclinical trials of AAV in primates. However, in these instances, liver toxicity emerged 4–5 days following drug delivery and was defined by the presence of elevated viral genome levels in the liver (>1000 viral genomes per cell) [151,152]. Researchers suggest that the mechanism of hepatotoxicity in primates, as opposed to humans, is probably associated with direct damage to hepatocyte membranes by AAV particles and/or activation of innate immunity [153].

During clinical trials and in medical guidelines, patients receive immunosuppressive treatment to avoid hepatotoxicity, the heightening of liver enzyme levels, and an increase in transgene expression [80]. The use of lower AAV doses is less efficient therapeutically but is accompanied by a low level of inflammation, which is successfully controlled with steroids.

#### 3.1.5. Dorsal Root Ganglia (DRG) Toxicity

The spinal ganglion consists of sensory neuron cell bodies that transmit information from sensory organs to the spinal cord [154]. Dorsal ganglia toxicity following administration of adeno-associated gene therapy has been observed in mice, primates, and other animal models. In general, this side effect was typically more prevalent when AAV was administered directly into the cerebrospinal fluid as opposed to intravenous injection [151,155,156]. Analysis of histological sections of animal samples showed the presence of degeneration and damage to nerve cells along with inflammatory infiltration of mononuclear cells [151,155,156]. Among clinical trials, spinal ganglion toxicity was seen at autopsy in a patient with familial amyotrophic lateral sclerosis (ALS) and mutations in the gene encoding superoxide dismutase 1 (SOD1) after intrathecal infusion of 4.2 × 10^14^ vg AAVrh10 containing anti-SOD1 microRNA (AAV-miR-SOD1). Approximately 3 weeks after receiving gene therapy, this patient developed neurological symptoms such as tingling sensations in the arms and pain in the left leg [157]. The mechanism of dorsal ganglia toxicity is currently unknown but it has been hypothesized that the death of neurons is associated with cellular stress due to overexpression of the transgene [158,159]. However, the possibility of the participation of the immune system in this process cannot be ruled out.

#### 3.1.6. Myocarditis

Myocarditis is believed to be another complication associated with immune-mediated toxicity of adeno-associated viral vectors. Myocardial infiltration of CD8+ T cells and electrocardiogram abnormalities consistent with myocarditis have been reported in baboons receiving a direct intramyocardial injection of AAV2-TNFRII-Fc to limit the progression of heart failure [160]. To date, among clinical studies, this complication has only been reported in patients receiving therapy for Duchenne muscular dystrophy. During a clinical trial of the drug SRP-9001 (AAVrh74 vector carrying minidystrophin) among 38 participants, myocarditis was recorded in one patient. Treatment with steroids prevented further progression of the adverse effect and impairment of systolic cardiac function [161]. Two patient deaths were also reported after administration of rAAV serotype 9 containing dCas9-VP64 at a dose of 1 × 10^14^ vg/kg and the fordadistrogen movaparvovec at a dose of 2 × 10^14^ vg/kg containing minidystrophin for the treatment of DMD. However, it was proposed that the patients’ deaths were linked to their innate immune response against the viral capsid in the myocardium, since there was no elevated organ effector T cell activity and no presence of neutralizing antibodies to AAV9 in the patients [162].

#### 3.1.7. Genomic Integration and Oncogenesis

As mentioned earlier, AAVs are non-integrating viral vectors and primarily persist in cells as episomes. However, some AAV DNA is still integrated into the host genome at a low frequency [96]. Thus, there is a potential for insertional mutagenesis as a result of the integration of the modified AAV genome. Several mechanisms exist for AAV integration. The first mechanism demonstrated in wild-type (WT) AAV is site-specific integration of the AAV genome into the AAVS1 locus involving the Rep 68/78 proteins encoded by WT-AAV. It was found that AAV vectors can be randomly integrated into the host genome, which probably occurs by double-strand breaks in the genomic DNA. This hypothesis is confirmed by the increased frequency of AAV genomes integrated into the host DNA upon induction of double-strand DNA breaks. Furthermore, AAV mostly integrates into regions of genomic instability that are susceptible to spontaneous breaks [163,164].

The oncogenic potential of recombinant AAV integration has been widely studied in recent years and still remains a controversial issue. The occurrence of hepatocellular carcinoma in mice due to AAV integration has been documented in many studies following systemic administration of the vectors, usually in the neonatal period [165,166,167,168,169]. Eighty percent of neonatal mice developed liver and lung tumors after injection of AAV9 expressing Hexb cDNA for correcting a Sandhoff disease [166]. In another study, tumors in various organs were developed in more than 20% of 44 animals that received the AAV vector either neonatally or 40 days after birth. In this case, hepatocellular carcinoma developed mainly after intrahepatic injection [167]. The majority of AAV integrations in neonatal mouse tumors were associated with the Rian locus and contributed to tumorigenesis due to disruption of gene expression. Also, the occurrence of hepatocellular carcinoma in newborn mice correlated with high doses and promoter strength of the administered AAV vector [170]. The vulnerability of newborn mice may be explained by early-life increased Rian expression and the presence of highly expressed genes that are preferential sites of AAV integration [171,172]. Conflicting outcomes have been observed in young mice (6–8 weeks) and adult mice following the administration of the viral vector. A number of studies have shown a lack of integration of the AAV genome into the host DNA [173,174], while other experiments have demonstrated the presence of AAV vector integration and an increased incidence of liver tumors [175]. Moreover, the presence of chronic liver disease and induction of hepatocyte proliferation increased the likelihood of hepatocellular carcinoma in adult mice by 95% [168].

Long-term studies in rats, dogs, primates, and clinical trials showed either a lack of AAV integration or rare cases of insertion that were random and not associated with tumor formation [98,176,177]. Thus, a study of liver biopsies obtained from non-human primates after administration of AAV5 for the purpose of treatment of acute intermittent porphyria showed a low frequency of integration of vector genomes and a random distribution of integration sites throughout the genome. Following the administration of 1 × 10^13^ vg/kg, 309 integration sites were observed, while a dosage of 5 × 10^13^ vg/kg resulted in the identification of 443 integration sites [176,177]. In clinical studies of AAV therapy for hemophilia B, observation of patients for more than 7 years did not reveal evidence of increased carcinogenic potential of AAV [178,179]. One patient was still diagnosed with hepatocellular carcinoma after receiving AMT-061 (etranacogene dezaparvovec). While this occurrence is concerning, it is likely that the AAV insertion was not the primary cause of cancer development. Instead, the patient’s extensive history of hepatocellular carcinoma risk factors, including genetic predisposition, a 25-year history of hepatitis C and B, evidence of non-alcoholic fatty liver disease (NAFLD), smoking, family history of cancer, and advanced age, are more likely to have contributed significantly to cancer development [180,181].

Although the frequency of integration of AAV vector genomes reported in clinical trials is low and their oncogenic potential is speculative, the presence of numerous clinical studies of AAV-based drugs highlights the need for further study of safety and potential genotoxicity. Additionally, limited information is available regarding the integration of AAVs into various tissues like the brain, muscles, or eyes. This aspect is crucial in assessing the safety profile of AAV-based drugs.

#### 3.1.8. Selecting the Administered Dose

The wide range of dosages currently used in clinical trials of adeno-associated vectors and the occurrence of deaths due to dose-dependent toxic side effects indicate that optimal viral vector dosing is challenging [80]. The situation is aggravated by the dependence of the optimal dosage on many factors, such as the AAV serotype, the route of drug administration, the type of promoter and transgene, and comorbidities in the patient. Additionally, determining the dosage of the delivered viral vector is also impacted by the presence of empty AAV capsids in the sample due to inefficient purification. However, data on the proportion of empty-to-full capsids at dosages employed in clinical trials are currently unavailable. A lower dose is considered safer in relation to toxicity and advantageous from a manufacturing perspective, but an excessively low dose runs the risk of inefficient target cell transduction. It is believed that more controlled dose-response studies in primates may help develop recommendations for the most effective and safe dosing regimens for future clinical trials. Selecting appropriate dosages for clinical trials in patients based on preclinical data is also a complex task. Currently, clinical dose selection for AAV gene therapy employs two primary methods: empirical and allometric scaling approaches, and mechanistic modeling [182]. Mechanistic models, unlike empirical models and allometric approaches, can incorporate human and animal physiology and anatomical parameters to improve dose transfer from preclinical to clinical trials. In addition, mechanistic models also allow the study of AAV kinetics, dynamics, and biodistribution in a dose- and serotype-dependent manner, thereby reducing the need for numerous preclinical studies [183,184].

#### 3.1.9. Tropism-Related Limitations

The occurrence of natural tissue tropism is an advantage of AAV over other viral vectors. Most of the serotypes are mainly employed for delivering the transgene to the liver, skeletal muscle, eye, and central nervous system [80,111]. However, AAV-mediated gene delivery to other tissues is challenging due to the relatively low transduction efficiency and tropism of natural serotypes for these tissues. For example, for the treatment of metabolic complications of obesity using AAV vectors, targeted delivery to adipose tissue is required. The use of broadly tropic serotypes for this purpose may result in reduced therapeutic efficacy due to toxicity and adverse immune-mediated reactions to transgene expression in non-target tissues [185]. Therefore, it is critical to develop AAV gene delivery vectors that are optimized for delivery to a specific tissue type. As with combating the adaptive immune response, strategies for searching for and creating alternative serotypes with improved tissue tropism are used for these purposes [186]. To improve AAV tropism for adipose tissue, a hybrid serotype Rec2 resulting from domain shuffling between AAV8 and rh20 (rhesus macaque-variant 20) was tested. Testing of this serotype showed higher transgene expression in both white and brown adipose tissue compared to natural serotypes AAV1, AAV8, and AAV9, which exhibit tropism for adipose tissue. In addition, off-target transgene expression in the liver after administration of the Rec2 hybrid serotype was lower compared to mice administered AAV1, AAV8, and AAV9 [187].

Modification of the AAV capsid can negatively affect the distribution and infectivity of the vector, so another challenge is to create systems for rapid screening of the biodistribution of new serotypes [188]. This task is complicated by the fact that the biodistribution in mouse models does not always coincide with the biodistribution in large animals and patients, as was demonstrated when testing capsids with tyrosine mutations in mice and dogs [189]. Therefore, the alternative capsids must necessarily be evaluated in large animal models such as non-human primates.

#### 3.1.10. High Cost

In 2022, AAV therapy (Hemgenix) for hemophilia B was the most expensive medication globally, with a price of USD 3.5 million per single administration. The high cost of AAV drugs is a result of the limited patient population affected by rare diseases and the need to compensate for the high costs of developing and manufacturing a biological product. In addition, the limited number of companies developing gene therapies for such diseases and the lack of competition determine the rather unregulated pricing of AAV therapies. The high cost of gene therapy is often a barrier to potentially life-saving interventions because patients cannot afford the expense themselves. Additionally, the expenses associated with these medications pose a significant financial strain on any nation’s economy. There are projections that the number of gene and cell therapy products available will reach 30–60 by 2030, and that by the end of 2034, 1.09 million patients will have been treated with gene therapy at a cost of up to USD 25.3 billion per year [190,191]. The current trends highlight the immediate necessity for introducing innovative and efficient payment and pricing systems to enhance the availability of AAV therapies [192].

#### 3.1.11. Ineffective Production Strategies

The high cost of rAAV gene therapy can be attributed primarily to the expensive manufacturing processes [76]. The main problem is the discrepancy between the low yield of the product and the high dose of the virus required for treatment. Plasmid transfection of adhesion culture is currently the most widely used method for generating high-titer and highly infectious AAV viral vectors [193]. However, the use of adhesion culture transfection for large-scale AAV production is economically prohibitive and challenging to scale up [194]. According to economic modeling studies of the AAV production process, alternative approaches to produce viral particles, such as suspension cultures and stable cell lines, are more cost-effective for generating large doses of the therapeutic compared to adherent cultures [195,196]. Although the transition from traditional methods to scalable technologies has already occurred in the field of AAV production for clinical use, there is still a plethora of problems that need to be solved.

Purification of AAVs is a critical and costly step, involving the removal of impurities such as empty or degraded AAV capsids, residual cellular proteins and nucleic acids of producer cells, leftover plasmid DNA, and components of the culture medium [197]. Ineffective removal of contaminants leads to decreased cell transduction and immune-mediated adverse reactions in patients [198]. Separating empty and full capsids presents a significant challenge due to their closely matched size and charge. Ultracentrifugation on a cesium chloride or iodixanol gradient is considered the gold standard for isolating the AAV fraction purified from empty capsids. Although this approach is operator-dependent, labor-intensive, and expensive when used on a large scale, it is still used in clinical and commercial production [193,199]. Anion exchange chromatography can also be used to differentiate between empty and full capsids. The degree of surface differentiation between empty and full capsids, as mentioned earlier, is small and also depends on the AAV serotype. This is manifested in the chromatogram by the presence of overlap between the peaks of empty and full capsids. Therefore, to achieve complete removal of empty capsids using this method, it is necessary to sacrifice a certain amount of full capsids [200]. Despite the loss of the target product, the use of anion exchange chromatography on an industrial scale is predicted to be more cost-effective compared to ultracentrifugation [196]. Additionally, to reduce the loss of the target product, the Multi-Column Counter-Current Solvent Gradient Purification technology has been suggested, incorporating a continuous filtration method using two columns instead of one. This method consists of continuous circulation of the overlap fraction, which is a mixture of empty and full capsids and various impurities, through a second column, thereby ensuring minimal loss of the viral vector [201,202].

The process of AAV replication and encapsulation in the cell may be an important factor limiting the large-scale viral stock production. It was shown that the number of empty capsids in the viral titer can vary from less than 50% to 98%, possibly stemming from improper biosynthesis and impaired assembly of viral particles [203]. Multi-omics technologies are actively employed to investigate the replication mechanism and metabolic pathways associated with virus production, as well as the metabolites that hinder these processes. Thus, a proteomic analysis of HEK293 cells that produce AAV5 uncovered proteins related to the endocytosis and lysosomal breakdown of viral vectors [204]. Multi-omics technology gathers and thoroughly analyzes data from genomics, transcriptomics, proteomics, and metabolomics to gain insights into molecular mechanisms and address challenges related to scaling up production and the low target product yield [202]. For example, differentially expressed genes, proteins, and metabolic pathways identified using multi-omics technologies may allow the development of strategies to optimize the culture medium to improve the performance of producer cells [204,205].

Batch-to-batch variability is a major concern in any bioprocess due to its complexity and variability, especially as production scales up. The increasing number of upstream and downstream platforms for producing adeno-associated vectors further complicates the regulation of production processes. At the same time, to obtain a safe and affordable biological product, it is necessary to maintain constant productivity, and it is advisable to control the quality of the product at each stage of production. Software solutions such as soft sensors can be used to solve this problem. Soft sensors are based on physical or machine learning models that can predict metrics of interest using other data fed to the measuring device [206]. This approach has been already successfully applied to real-time monitoring of critical process variables to improve the quality and speed of monoclonal antibody production, and researchers believe that this technology holds promise for the generation of viral vectors [207].

Thus, improving technologies for the purification of AAV vectors and introducing innovative technologies to increase product yield can significantly reduce the cost and improve the safety of gene therapy with adeno-associated vectors in the future.

### 3.2. Baculoviral Vector

Baculoviridae is a family of enveloped dsDNA viruses that are highly specific for their natural insect hosts, such as arthropods and lepidopterans, at the larval stage. Baculoviruses have a large genome size, ranging from 80 to 180 kb. The genome is packaged in a rod-shaped nucleocapsid surrounded by a membrane and measuring approximately 30–60 nm in diameter and 250–300 nm in length [208]. Autographa californica multicapsid nucleopolyhedrovirus (AcMNPV) and Bombyx mori MNPV (BmMNPV) represent the most extensively studied strains and are most commonly used in gene therapy and biotechnology [209,210].

The life cycle of a baculovirus involves two viral forms: budded-virus (BV) and occlusion-derived viruses (ODVs). They contain the same genetic information but solve different problems and occupy different subcellular localizations. Outside the host, virions form occlusion bodies (OBs), which consist of polyhedrin proteins that protect the viruses from harsh environmental conditions. Once occlusion bodies reach the insect’s gut, changes in pH lead to the breakdown of the protein matrix, leading to the release of occlusion-derived viruses. While ODVs are responsible for the primary infection, budded-viruses (BVs) are second-type virions that are produced by infected cells and are surrounded by a cell membrane [211,212]. In laboratory and biotechnological settings, BVs are commonly used, whereas ODV production is prevented by deleting the polyhedrin protein gene from the AcMNPV genome. The production of recombinant baculoviruses can be carried out in insect cell lines derived from the ovaries of Trichoplusia ni and Spodoptera frugiperda, such as Sf9, Sf21, and High Five [212,213]. Importantly, baculovirus is a non-integrating vector and poses no risk of insertional mutagenesis [214].

Because of its ability to mediate post-translational modifications and better folding of mammalian proteins compared to bacteria, baculovirus is widely recognized in academic and industrial laboratories as an efficient system for producing recombinant proteins. This recombinant protein production system is called the baculovirus expression vector system (BEVS) [210]. BEVS achieves a high level of protein expression because it triggers the shutdown of all endogenous promoters in infected cells at a late stage and promotes transcription from the viral polyhedrin and p10 promoters [215]. Many biotechnological preparations of enzymes, hormones, and virus-like particles are created using BEVS technology [209,210,216]. For example, this expression system formed the basis for the production of the vaccine against human papillomavirus types 16 and 18 Cervarix, which was approved by EMEA and FDA and consists of virus-like particles [217]. Also, the baculovirus vector expression system was introduced in 2002 as an effective strategy for scaling up the production of AAV in gene therapy [218]. The BEVS system developed Glybera^®^, the first AAV drug approved for treating lipoprotein lipase deficiency. In addition to the GOI, promoter, terminator, and various gene regulatory elements, the large packaging capacity of the baculovirus allows additional genes encoding various proteins and subunits of the multiprotein complex to be cloned into the recombinant baculovirus genome. This feature formed the basis of the MultiBac expression system. The application of this technology enables the analysis of multiprotein complex structures, as evidenced by research on the assembly mechanisms of a transcription factor complex and the structures of G-protein-coupled receptors (GPCRs) [219].

While baculoviruses primarily infect insects and cannot reproduce in vertebrate cells, they can enter mammalian cells. Baculoviruses can transduce common cell lines such as HeLa, Huh-7, HepG2, HEK293, and MSCs, and human nerve cells [220]. The surface glycoprotein GP64 plays an important role in the processes of virus attachment, internalization, and exit from endosomes of both mammals and insects [220]. After the discovery of the ability of baculovirus to transduce mammalian cells, the use of baculovirus particles as a potential vector for gene therapy began to rapidly develop. The system, consisting of a baculovirus carrying a mammalian promoter and capable of efficiently transducing vertebrate cells, was called “BacMam”, and it is used in many in vitro and in vivo applications, benefiting from some features of baculoviral gene delivery [221,222,223,224]. First, unlike AAVs and retroviruses, baculoviruses have a higher transgenic potential and can support inserts exceeding 38 Kb in size [225]. Similar to MultiBac, the MultiBacMam system was developed to deliver multiple GOIs simultaneously into mammalian cells and tissues. This technology has shown potential in facilitating the delivery of CRISPR-Cas gene editing components, and as a tool for analyzing protein–protein interactions (PPIs) by fluorescence complementation [226,227,228]. In addition, the baculovirus has a flexible viral envelope and capsid that simply increases in size in proportion to the insertion of heterologous DNA [229]. Second, the baculovirus can effectively transduce both dividing and non-dividing cells without having toxic effects or inhibiting cellular growth, even at high MOIs [220,230,231]. The transduction efficiency of the baculovirus vector compared with the adenoviral vector was assessed in vitro in liver cell lines (Huh7 and HepG2), COS7, and HeLa and was similar to that of the adenovirus with a markedly less cytopathic effect. Baculoviruses cannot reproduce in mammalian cells, which eliminates the possibility of creating replication-competent viral particles through recombination in the host body. This characteristic significantly contributes to the safety of baculovirus vectors [232]. Another advantage of baculoviruses, compared with viral vectors such as AAV and adenoviruses, is the absence of pre-existing immunity, which makes transduction with baculovirus vectors more stable and safe. In addition, through genome engineering and pseudotyping, the tropism of baculovirus vectors can be tuned [220,233].

The availability of cost-effective commercial platforms for the efficient production of recombinant baculoviruses has also increased the appeal of this system. Most technologies for producing recombinant baculoviruses carrying GOI are based on two strategies [234,235]. The first mechanism is a process of homologous recombination within insect cells between a bacmid (the baculovirus genome designed to replicate in *E. coli*) and a plasmid, which is an expression cassette with a promoter, a GOI, a transcription termination signal, and two regions that enable recombination with the bacmid. In this case, the bacmid carries a truncated gene necessary for the reproduction of the baculovirus, which prevents the production of wild-type viruses. A recombinant baculovirus can only replicate in insect cells after the truncated gene is successfully restored through recombination. The flash BAC platform (Oxford Expression Technologies, Oxford, UK) is based on the described approach. Another strategy is the basis of a commercially available Bac-to-Bac expression system (Invitrogen Inc., Carlsbad, CA, USA). It comprises a site-specific transposition between a bacmid and a plasmid cassette carrying GOI and flanked by Tn7 transposition sites. Transposition of the Tn7 transposon occurs in *Escherichia coli* DH10Bac cells, which carry the bacmid sequence and a helper plasmid encoding the Tn7 transposase. Various methods for assembling multigene constructs in the baculovirus genome have also been developed, which are based on molecular cloning technologies such as Cre-lox recombination (MultiBac) [236], Golden Gate (GoldenBac) [237], and Gibson assembly (biGBac) [238].

Despite the advantages of baculoviruses as a system for gene delivery, the development of in vivo gene therapy based on baculovirus vectors lags significantly behind leading vectors such as AAV, adenoviral, and lentiviral vectors. This is primarily due to limiting factors such as complement activation in response to the injection of baculovirus into the bloodstream, transient gene expression, and the fragility of the baculovirus vector.

#### 3.2.1. Activation of the Complement System and Immune Response

The discovery of the inactivation of baculovirus upon systemic administration in vivo due to activation of the complement system has become a significant barrier limiting the transition to further testing of baculovirus vectors in large animal models. It has been established that both classical and alternative pathways are involved in complement activation in response to baculovirus vectors. Hoare et al. reported that the survival of baculovirus vectors is only 25% in human serum with a complete absence of classical complement activity caused by absolute C1q deficiency. Inhibition of the classical and lectin pathways with EGTA increased vector survival by 30%, whereas complete neutralization of all complement activity with EDTA restored 100% survival. However, it was shown that the lectin pathway does not play a significant role in this process [239]. Further studies using an ex vivo human blood loop model showed that the mechanism of complement activation in response to baculovirus particles involves their opsonization by both IgM and C3b [240].

Several strategies prevent inactivation of the baculovirus vector by the complement system reducing the immune response. These include delivery to immune-privileged sites, ex vivo transduction, the use of pharmacological complement inhibitors, and engineering the baculovirus surface [241].

Baculovirus vectors can trigger a robust innate immune response and induce the production of significant amounts of IFNα, IFNβ, and pro-inflammatory cytokines [242,243,244]. TLR9 recognizes CpG motifs in the baculovirus genome, which contributes to the immune response [245]. In addition to activation of the TLR9/MyD88 pathway, the adapter protein STING has been identified as a central mediator of interferon synthesis in response to baculoviruses [246]. Although recombinant baculovirus vectors effectively enter the nucleus of mammalian cells for transgene expression, baculovirus particles are often retained in the cytoplasm and remain available for interaction with cellular DNA sensors. It has been demonstrated that the cyclic GMP-AMP synthase (cGAS) DNA sensor, upon recognition of the baculovirus genome, activates the STING-TBK1-IRF3 cascade, stimulating the production of type I IFN and enhancing the production of type III IFN. Moreover, an alternative cytosolic sensor called DNA-dependent protein kinase (DNA-PK) was found to activate STING independently of cGAS in epithelial cells. The DNA-PK-dependent pathway is responsible for IFN-λ1 production in human epithelial cells [243]. The production of IFN-I can negatively affect the expression of the transgene delivered by the viral vector by preventing the transit of the virus into the cell, increasing intracellular pH, etc. In the case of the baculovirus vector, it was experimentally proven that the antiviral response suppresses the transgene expression mainly through the cGAS-STING pathway [243]. Although the induction of an innate immune response allows the effective use of baculovirus as immunomodulators and adjuvants for vaccines, in the case of gene therapy, this response can greatly affect the effectiveness of gene therapy.

The most promising strategy for avoiding activation of the immune system is to modify the surface of baculovirus particles by chemical and biological means. Polymers such as polyethyleneimine (PEI) and polyethylene glycol (PEG) are widely used to chemically protect vectors from recognition by blood components [247,248]. It was demonstrated that modification of baculovirus with PEI 25 kDa prevented the reduction in transduction efficiency and resulted in improved baculovirus stability in 10% serum compared with unmodified virions [247]. Coating the baculovirus, based on electrostatic interaction, with another polymer, PEG (Mw 5000), increased transduction efficiency in vitro and in vivo when delivered to the brain and lungs of mice [248]. Because polymers can be cytotoxic and negatively affect virus infectivity, an important caveat of this approach is the need to carefully optimize the ratio of polymer and viral particles. The surface of the baculovirus vector can also be modified using a genetic engineering approach. Pseudotyping is a process in which the natural envelope proteins of a virus are replaced with the surface proteins of another virus. Baculovirus vectors are commonly pseudotyped with the vesicular stomatitis virus glycoprotein (VSV-G), which is similar to retroviral and lentiviral constructs [249]. The VSV-G pseudotyped baculovirus vector exhibits resistance to inactivation by human, rabbit, guinea pig, rat, hamster, and mouse serum compared to unmodified baculovirus, the extent of which varies depending on the origin of the serum [250]. In addition to VSV-G, the surface display of complement-regulating proteins on baculovirus virions has been tested to reduce complement-mediated inactivation of baculoviruses. The presence on the surface of the recombinant baculovirus of factors such as a decay-accelerating factor (DAF), membrane cofactor protein (MCP), and C4b-binding protein helps protect the baculovirus from attack by the immune system.

Intraportal administration of high doses of the unmodified baculovirus vector was fatal in mice. Exposure of the DAF factor to the surface of the baculovirus increased the survival rate of mice [251]. Although pseudotyping the surface of a baculovirus vector increases its stability in blood serum, this approach does not completely solve the problem of inactivation of viral particles by complement. This emphasizes the need for enhanced surface designs and their optimization [241].

In addition to pseudotyping, the survival of two-thirds of mice treated intraportally with high doses of baculoviruses was facilitated by the injection of soluble CR1 (sCR1), which binds to C3b and C4b [239]. Compstatin has also been demonstrated to have an inhibitory effect on complement activation in an ex vivo whole-blood model, making this molecule a promising candidate for clinical guidelines using recombinant baculoviruses [240]. Another way to prevent baculovirus inactivation by the complement is to use complement inactivators before delivering the viral vector in vivo.

Delivery of the baculovirus vector to immune-privileged sites such as the brain, eye, and testicles may also offset vector inactivation by the complement system. The ability to deliver a transgene to the eye using baculovirus particles has been demonstrated in mice, rats, and rabbits [252,253,254]. Thus, injection of the baculovirus BacVEGF-D into the vitreous body caused the expression of vascular endothelial growth factor D in the inner retina, photoreceptor cells, and retinal pigment epithelial cells [253]. However, when comparing AdV, AAV, LV, and baculovectors encoding GFP, intravitreal injection of the baculovirus vector demonstrated the lowest percentage of GFP-positive cells as well as the strongest immune response comparable to AdV injection [254]. High BV-mediated transduction efficiency was observed in normal astrocytes in vitro and in vivo, as well as in human and mouse glioma cells and neurospheres in vitro and in vivo. The efficient delivery of the baculovirus construct to the brain, along with its low neurotoxicity, makes it a promising vector for gene therapy treatments for brain tumors [255]. Attempts to deliver recombinant baculovirus vectors into mouse testicular tissue have demonstrated a lack of spermatocyte transduction, suggesting that baculoviruses can be used without the risk of germline transmission [256,257].

An alternative immune evasion strategy that does not involve baculovirus surface modification is ex vivo gene delivery. The use of bone marrow-derived mesenchymal stem cells (BMSCs) transduced with BMP2- or VEGF-encoding baculovirus ex vivo accelerated the healing of large femoral bone defects and improved the quality of the regenerated bone [258]. The baculovirus vector was also used for CRISPRa (CRISPR activation technology)-mediated activation of mitochondrial uncoupling protein 1 (UCP1) in adipocytes to stimulate thermogenesis and subsequent subcutaneous transplantation of modified adipocytes in Matrigel [259].

In addition, recombinant BmMNPV is more resistant to complement inactivation by human serum compared to the widely used baculovirus AcMNPV. Thus, this characteristic makes the baculovirus BmMNPV preferable for in vivo gene delivery [260].

#### 3.2.2. Transient Gene Expression

Baculovirus vectors are known for their temporary expression of the transgene. Typically, transgene expression in vivo and in vitro decreases on day 7 and lasts for no more than 14–21 days [255,261,262]. The short period of transgene expression in vivo is partly due to the inactivation of the baculovirus vectors by the activated complement system. Therefore, the application of the abovementioned complement control methods can effectively prolong gene expression in vivo. For example, pseudotyping of VSV-G baculovirus particles extended gene expression to 35 days in BALC/c and C57BL/6 mice and to 178 days in DBA/2J mice [249].

However, the temporary expression of the transgene can also be explained by the physiological characteristics of the baculovirus delivery system. As described above, the DNA of retroviral, lentiviral, and adenoviral vectors can persist in the nucleus in an integrated or episomal form for a long time. In contrast to these viral delivery systems, DNA from baculoviral vectors has been shown to persist in the nuclei of transduced mammalian cells for only 24–48 h [232].

A hybrid baculovirus vector based on the Sleeping Beauty (SB) transposon system was developed to prolong the duration of transgene expression. This baculovirus expresses the SB transposase and contains a transgene cassette flanked by an inverted/direct repeat (IR/DR). After entering the host cell, this system ensures that the transposon is excised and integrated into the host chromosome. This hybrid system effectively transduced the HepG2 cell line and ensured in vitro transgene expression within 63 days of observation because of transgene integration and persistence [263]. Long-term transgene expression for at least 2 months was also observed after intravitreal and intramuscular injections of the SB hybrid baculovirus system [263,264].

In addition, Wang Z et al. combined the SB transposon system and pseudotyping with the DAF protein to develop a hybrid bivalent baculovirus vector. The use of this construct demonstrated both increased resistance to complement and stable transgene expression in vivo and in vitro, where eGFP expression was maintained for at least 90 days [265].

#### 3.2.3. Fragility of Baculovirus Vector

A common problem with enveloped viruses, such as baculoviruses and retroviruses, is the fragility and vulnerability of the viral envelope to mechanical shear force [266]. This feature determines the low stability of these viruses. As already mentioned, the glycoprotein GP64 is located on the surface of the baculovirus, which is necessary for the transduction of the baculovirus vector into mammalian cells. During ultracentrifugation to purify baculovirus particles, the increased mechanical shear force results in loss of GP64 and infectivity due to damage to the viral envelopes. In addition, the stability of baculoviruses depends on the temperature. The half-life of baculoviruses at 27 °C is about a week, whereas at 37 °C, it is 7–8 h [267]. The problem of baculovirus inactivation by serum complement, coupled with fragility and heat sensitivity, seriously limits the application of baculovirus vectors for in vivo delivery.

If the primary constraint of the baculovirus vector, namely, immune system activation, can be addressed, its safety and high cloning capacity may enable it to rival retroviral vectors in ex vivo gene therapy applications. The activation of intracellular innate immunity can be neutralized by small-molecule inhibitors of the STING signaling pathway, such as poxin P26 [268]. Simultaneously, for effective systemic gene delivery in vivo, serious work is required to develop suitable baculoviral vectors that are resistant to the influence of the complement system.

## 4. RNA-Based Viral Vectors for Gene Therapy

### 4.1. Gamma-Retroviral and Lentiviral Vectors

**Figure 4:**
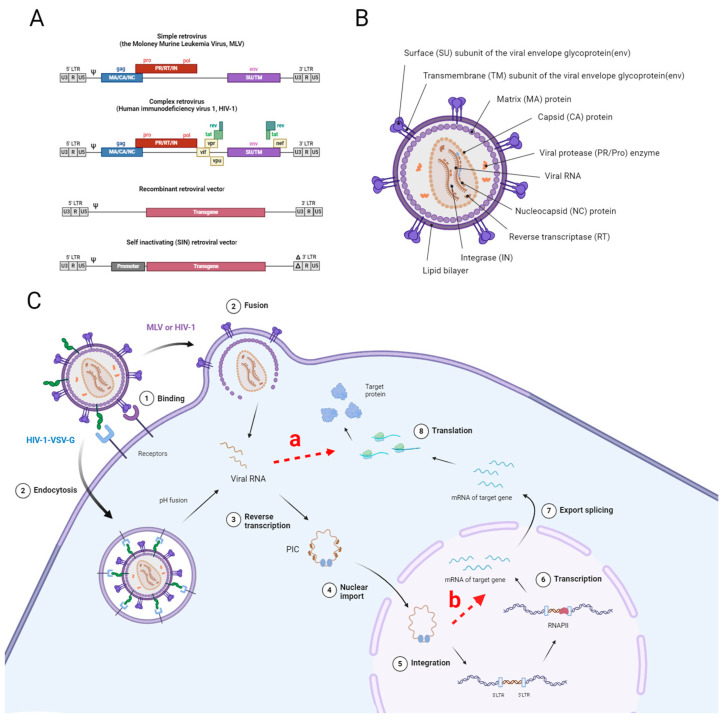
(**A**). Schematic representation of the genome of simple and complex retroviruses, a recombinant retroviral vector and a SIN retroviral vector. All retroviruses have four main genes in their genome: gag, pro, pol, and env. These genes encode capsid proteins, viral protease, reverse transcriptase, and integrase, as well as viral envelope proteins. The genome of certain lentiviruses, like HIV-1, comprises several regulatory and accessory genes, including tat, rev, vpr, vpu, nef, and vif. The retrovirus genome is encircled by 5‘- and 3‘-long terminal repeats (LTRs), which include U3, R, and U5 sequences. Self-inactivating (SIN) vectors have a deletion in the 3‘LTR of the viral genome that is transferred into the 5‘LTR after one round of reverse transcription. (**B**). Structure of retrovirus particle using HIV-1 as an example. (**C**). Scheme of transgene delivery into a cell using a retroviral vector: (1) retroviruses bind the membrane, and next, depending on the envelope protein, the retroviral vector (2) enters the cell either by fusion with the cell membrane (in the case of viral vector based on HIV-1 or MLV) or through endosomes (in the case of viral vector based on pseudotyped HIV-1 with VSV-G). After the genomic material is released from the viral particle, the viral RNA is reverse-transcribed by the virus-encoded reverse transcriptase enzyme (3). If reverse transcription is absent, the mRNA may be subject to immediate translation (a). The resulting pre-integration complex (PIC) (in the case of HIV-1-based viral vectors) enters the nucleus through a nuclear pore (4), while MLV-based viral vectors require the nuclear membrane to disintegrate during mitosis to gain access to the cell’s genomic material. After integration (5), transcription of the target transgene begins (6). In case of blocking integration into the cellular genome, the genetic material of the retroviral vector is stored in the form of episomes, which can persist for a long time in non-dividing cells (b).

Retroviruses are RNA viruses 80–120 nm in size, surrounded by an envelope and containing two copies of plus-strand RNA [269,270]. The family Retroviridae includes the following kinds: alpha (Rouse sarcoma virus, RSV), beta (Mouse mammary cancer virus, MMTV), gamma, delta (Human T-cell leukemia virus type 1, HTLV-1), epsilon (The Walleye dermal sarcoma virus, WDSV), lenti- (Human immunodeficiency virus 1, HIV-1), and spumaviruses (Human and Simian foamy viruses). The genome of all retroviruses contains 4 main genes—gag, pro, pol, and env, which encode capsid proteins, viral protease, reverse transcriptase and integrase, and viral envelope proteins. Lentiviruses, such as HIV-1, also have additional regulatory and accessory genes in their genome: tat, rev, vpr, vpu, nef, and vif. After entering the host cell, the viral RNA genome is converted by reverse transcriptase into double-stranded DNA, which is then integrated into the host cell genome by viral integrase. The retrovirus genome is surrounded by 5‘- and 3‘-long terminal repeats (LTRs), which consist of U3, R, and U5 sequences and function as promoters [269,270] (Figure 4A,B).

Retroviral vectors have many features that make them attractive vectors for gene therapy. Compared to AAV, retroviral vectors have a greater packaging capacity (up to 8 kb), which significantly expands the scope of their application. An important characteristic of retroviral-based vectors is their ability to integrate into the host cell DNA and provide long-term transgene expression (Figure 4C) [271]. In addition, the presence of an envelope in the form of a cellular bilayer determines the low immunogenicity of retroviral vectors compared to delivery systems based on adenoviruses and AAV [272]. The broad tropism of most retroviral vectors is achieved by pseudotyping using different membrane proteins, allowing viral particles to infect different cell types [273,274,275], due to their ability to penetrate an intact nuclear membrane, lentiviral vectors, unlike gamma-retroviral vectors.

Among the first viral vectors developed in the 1980s and 1990s were gamma-retroviral vectors derived from murine leukemia virus [276]. Today, Tecartus and Yescarta are authorized CAR-T treatments for gamma-retrovirus-related tumors available in the market. However, despite their initial success, the number of clinical trials involving MLV-based vectors has decreased significantly. At the same time, the number of clinical studies involving lentiviral vectors has increased [273]. In 2018, more than half of the clinical trials in the US relied on the use of LV to generate CAR T cells [277]. In addition, drugs based on lentiviral vectors, Kymriah, Abecma, Breyanzi, and Carvykti, have been approved for the treatment of malignant neoplasms. In 2024, the FDA approved Libmeldy for the treatment of metachromatic leukodystrophy, which is based on autologous CD34+ stem cells transduced ex vivo with a lentiviral vector encoding the human ARSA gene. Due to the improved safety profile of lentiviral vectors, and their ability to transduce non-dividing cells, they have replaced gamma retroviruses as the leading delivery system for the clinical application of ex vivo gene therapy.

At the same time, due to the complex technological process, ex vivo CAR-T therapy involves many risks for the patient and also requires huge costs from the healthcare system. These difficulties can be overcome by generating CAR T cells in vivo by administering a viral vector [278]. In vivo generation of CAR T cells has been successfully demonstrated by several research groups in mouse models [279,280,281]. However, the widespread application of these vectors in both in vivo and ex vivo gene therapy is hindered by certain drawbacks associated with retroviral vectors, which are discussed below.

#### 4.1.1. Insertional Mutagenesis

One major limitation of lentiviral and other retroviral vectors, which affect the safety of these delivery systems, is insertional mutagenesis. This results from the integration of the vector into the host cell’s genome.

MLV-based vectors were first successfully used in the clinic to treat patients with X-linked severe combined immunodeficiency (X-SCID) using ex vivo modified hematopoietic stem cells (HSCs). However, after using gamma-retroviral vector therapy, 5 out of 20 patients developed leukemia [282]. Subsequent analysis of genetically modified cells showed that the presence of tumor transformation correlated with the integration of MLV vectors near genes involved in cell proliferation (LMO2, BMI1, CCND2) [283]. The development of acute leukemia induced by integration near LMO2 has also been observed in patients receiving gene therapy with gamma-retroviral vectors for the treatment of X-linked chronic granulomatous disease (X-CGD) and Wiskott–Aldrich syndrome (WAS) [284,285]. Early gamma-retroviral vectors are characterized by the presence in their genome of strong enhancers located in long terminal repeats. Further studies showed that the observed insertional mutagenesis was a consequence of the insertion of these strong gamma-retroviral vector enhancer elements into the host DNA [286]. Following the discovery of tumors caused by gamma-retroviral vectors, the field of retroviral gene therapy has shifted its attention to lentivirus vectors and has stimulated research to improve the safety of gamma-retroviral gene therapy vectors.

In the instance of lentiviral vectors, early generations of vectors in mouse models showed oncogenic transformation as a result of insertional mutagenesis [287]. Although tumorigenesis has not been reported in numerous clinical studies of lentiviral vectors, cases of benign clonal expansion of modified cells have been reported [288]. Thus, during treatment with modified CAR-T cells in a patient with chronic lymphocytic leukemia, the descendants of one modified cell made up the majority of the CAR-positive population and contained a lentiviral vector integrated into the TET2 gene. This integration led to the termination of TET2 RNA splicing and a decrease in DNA modifications catalyzed by the TET2 protein. However, in this case, insertional mutagenesis, carried out by inactivating the TET2 gene, apparently only promotes the proliferation of CAR-T cells and effective therapy [289,290]. Clonal expansion has also been observed when lentiviral vectors deliver wild-type copies of the beta-globin gene into bone marrow stem cells to correct beta-thalassemia [291]. This process has been associated with the integration of a viral vector into the HMGA2 gene, which is often found in various types of benign tumors. Cell proliferation was associated with overexpression of HMGA2 caused by replacement of the 3‘ end of HMGA2 mRNA and removal of the target site of regulatory microRNA due to insertional mutagenesis [286,292]. Thus, integration of retroviral vectors can influence pro-oncogenes and lead to cell proliferation through various mechanisms, such as gene activation by integration of enhancer sequences, triggering gene activation by altering the 3‘ end of mRNA, and causing gene inactivation by disrupting its coding sequence [286].

The development of self-inactivating (SIN) vectors has significantly improved the safety profile of both gamma-retroviral and lentiviral vectors. SIN vectors are characterized by the absence of a sequence of strong promoter and enhancer elements in the LTR, while the transgene is expressed by an internal promoter. Meanwhile, several research studies indicate that the potential for insertional mutagenesis remains elevated when utilizing SIN vectors [293,294]. In addition, the use of strong internal promoters and enhancer elements within SIN vectors can lead to the activation of neighboring genes after integration [295,296].

Gamma retroviruses and lentiviruses have different preferences when choosing integration sites in the host genome [297]. MLV has an affinity for transcription start sites (TSSs), enhancers, and promoter regions, while HIV-1 prefers to integrate into actively transcribed genes [298,299]. Although each retrovirus typically has particular integration sites, they also have a tendency to integrate in areas beyond these specific regions [300]. In addition, in many retroviral families, the integration into genomic elements is influenced by integration cofactors [301]. To reduce insertional mutagenesis, approaches are being developed to target retroviral vector integration at specific sites in the host DNA. The development of MLV-based retroviral vectors independent of the BET cofactor resulted in reduced integration into TSSs and CpG islands, along with a lower incidence of tumorigenesis. However, in some cases, MLV integration was still observed in regions associated with tumorigenic genes, suggesting the need for additional modification of the vectors [302]. More detailed information about targeting the integration of retroviral vectors can be found in the following review [303].

To mitigate the increased risk of insertional mutagenesis, non-integrating lentiviral vectors (NILVs) have also been developed through the introduction of mutations into the integrase gene or by altering the attachment sequences of the LTRs to prevent recognition of viral DNA by this enzyme. NILVs can effectively express transgenes in non-dividing cells due to the presence of vector DNA stored in episomal form. Whereas, in dividing cells, only transient expression is available due to the dilution effect of episomes. Another disadvantage of NILV is reduced transgene expression compared to unmodified lentiviral vectors. Meanwhile, there are approaches aimed at overcoming this limitation, including the use of strong promoters/enhancers or the addition of HIV-1 auxiliary proteins (Vpr or Vpx) to the construct. Although loss of NILV episomes limits the use of these vectors, transient expression of NILV is an advantage in applications such as vaccination, cancer immunotherapy, cellular differentiation, and template delivery for homologous recombination [304].

Integration and long-term expression of the transgene, characteristic of unmodified lentiviral vectors and gamma-retro viruses, prevents the safe use of these viral vectors for delivery of the CRISPR-Cas9 system. This is associated with a high risk of off-target mutagenesis due to the stable expression of Cas9 and sgRNA [305]. According to research, NILVs are highly promising for delivering CRISPR-Cas9 systems as they offer comparable effectiveness in editing target DNA and a lower percentage of unintended mutations [306].

#### 4.1.2. Formation of Replication Competent Viral Particles

The unintentional generation of replication-competent retroviruses in host cells is another problem associated with the use of retroviral vectors [307]. Recombination of a lentiviral vector with wild-type HIV in HIV-infected individuals can lead to the reconstruction of a pathogenic virus capable of replication and development of infection. This procedure presents a risk not only to patients but also to laboratory staff who have direct contact with biological objects [308].

The number of recombination events that are required to assemble a replication-competent viral genome, and the number of genes important for replication and virulence that are removed from the vector system determine the risk of generating replication-competent lentiviral vectors. In this regard, the creation of recent generations of vectors has significantly increased the safety of retroviral delivery systems, by dividing the transgene and the genes required for packaging into four or more plasmids, and by strictly regulating or removing the necessary virulence and replication genes [308]. To this day, there have been no reported instances of the formation of replication-competent viral particles in any clinical trials. However, the possibility of this occurrence has not been fully eradicated [309,310].

#### 4.1.3. Limitations of Pseudotyping

Limitations of natural tropism of retroviral vectors can be overcome by pseudotyping. The most common approach for pseudotyping retroviral vectors is the use of Vesicular stomatitis virus G (VSV G) protein. In addition to providing a broad cellular tropism, the use of this glycoprotein increases the stability of viral particles, which is a huge advantage in the production and purification of targeted viral vectors [273]. On the other hand, there are some difficulties that arise when using VSV-G for the purpose of pseudotyping retroviral vectors. VSV glycoprotein G is bound and inactivated by complement, which may prevent the effective use of VSVg-pseudotyped retroviral vectors in vivo [311,312]. In addition, the VSV-G glycoprotein is cytotoxic when expressed stably in cells, making it difficult to develop stable producer cell lines for the production of retroviral vectors [313]. VSV-G pseudotyping’s broad tropism is not a drawback when targeting the liver for in vivo gene therapy. It contributes to the successful in vivo use of lentiviral vectors containing blood coagulation factor IX for correcting hemophilia in dogs due to the vector’s natural biodistribution [314]. However, for other gene therapy targets, the broad cellular tropism provided by VSV-G glycoprotein may be responsible for off-target side effects when administered in vivo.

Developing and testing new pseudotyping retroviral vectors is underway to address the limitations associated with VSV-G pseudotyping. The use of alternative virus envelope proteins demonstrates many differences in cellular tropism, titer, virus particle stability, and transduction efficiency compared to “classical” pseudotyping [274,275]. Thus, pseudotyping of lentiviral vectors with rabies virus glycoprotein can be used for targeted delivery of the transgene into neurons [315]. Pseudotyping with measles virus glycoproteins makes lentiviral vectors attractive tools for in vivo gene therapy targeting the immune system due to their limited tropism predominantly for lymphoid cells [316]. However, these pseudotyping approaches are also not without drawbacks and face individual limitations. For example, the presence of specific immunity against the measles virus resulting from vaccination or natural infection potentially precludes systemic administration of MV-G pseudotyped vectors to patients [274].

### 4.2. Foamy Viral Vectors

A promising alternative to the above viral vectors for gene therapy in vivo are vectors based on Foamy viruses (FVs), which belong to the spumavirus genus of the Retroviridae family [317]. These viruses mainly target non-human primates. In humans, infection with FVs is rare and usually occurs as a result of zoonotic transmissions, often affecting individuals who have close contact with these animals [318]. In addition to low seroprevalence in humans, viral vectors based on FVs have other advantages that set them apart from other retroviral vectors. The most important advantage of foamy viral vectors is the reduced risk of insertional mutagenesis, which is determined by the safer integration profile of these vectors compared to lentiviral vectors [319,320]. Foamy viral vectors (FVVs) also have broad tropism due to penetration into the cell using heparan sulfate, which is present on the surface of various types of cells [321]. Foamy viruses have the largest genome among mammalian retroviruses, allowing the use of vectors based on them to deliver longer transgene cassettes (approximately 12 kb). Thus, this vector was tested for delivery of the full-length version of dystrophin (11 kb) into cells of muscle origin. The titer of the viral vector decreases with increasing FVV genome size. However, it was demonstrated that the decline in the viral titer of FVVs after dystrophin delivery did not surpass 100-fold and still facilitated effective transduction of the cell line [322]. FVVs are also resistant to inhibition by the complement system, which makes possible stable delivery of vectors to target organs [323].

FVV is a promising tool for ex vivo therapy of hereditary diseases [324,325]. When transducing CD34+ peripheral blood cells of dogs, the efficacy of these particles was comparable to the lentiviral vector, and stable long-term (>700 days) expression was observed after transplantation into animals [324]. In addition, vectors based on Foamy viruses are actively being tested in gene therapy in vivo [323,326]. Thus, the FVV was successfully used in a preclinical model of SCID-X1 in dogs in vivo and showed efficacy comparable to clinical trials of γ-RVV and LV vectors in ex vivo gene therapy in patients suffering from SCID-X1 [323].

A significant disadvantage of FVV is the dependence of integration on cell division. At the same time, viral particles that enter the cell can persist around the centrosome for at least 30 days while awaiting stimulation of cell division [327]. In addition, technologies for pseudotyping these vectors have not yet been developed. This task is further complicated by the differences in the life cycle of Foamy viruses compared to gamma and lentiviruses [328].

## 5. Oncolytic Viruses

Because of the immediate response of the innate and adaptive immunity to in vivo administration and the transient expression of transgenes, most RNA and DNA viruses such as adenovirus and HSV-1 are not preferred for gene delivery. However, due to the specified characteristics and genetic engineering modifications to maintain the ability to replicate, these viruses are used in gene therapy as promising platforms for developing oncolytic viruses (OVs) [329]. The approval of oncolytic drugs Oncorine (H101), based on the adenovirus, Imlygic (T-VEC), and Delytact (G47Δ), derived from the herpes simplex virus, for the treatment of malignancies is fueling interest in this field. In addition, there are currently more than 200 registered clinical trials investigating the therapeutic use of various OVs as single agents or as part of combination therapy [330,331,332].

The main antitumor mechanism of OV involves the infection, replication, and lysis of cancer cells. Due to the loss of the antiviral response in tumor cells, oncolytic viruses act selectively and leave healthy cells unharmed [333]. In addition, the presence of aberrant signaling pathways in tumor cells, such as p53, interferon (IFN), retinoblastoma (Rb), and RAS/RAF/MEK/ERK pathways, influence the breakdown of viral defense mechanisms and enhance the effect of certain OVs [334,335]. An alternative mechanism for the therapeutic effect of OVs is the stimulation of immune-mediated antitumor effects. Direct oncolysis results in the release of tumor-associated antigens (TAAs), tumor-associated neoantigens (TANs), and damage-associated molecular patterns (DAMPs). This process initiates the activation of the innate response and the transformation of an immunologically “cold” tumor into a “hot” tumor. This phenomenon occurs predominantly because of the recruitment and migration of neutrophils and macrophages, and the activation of NK cells and DCs thanks to the release of cytokines and chemokines in response to PAMP/DAMP. Also, the high availability of tumor antigens stimulates the adaptive immune response by activating dendritic cells to present antigens and recruiting both virus- and tumor-specific CD4+ and CD8+ T cells to the tumor [336]. Activated T cells play a role in eliminating tumor cells not only within the solid tumor but also in distant tumors and metastases, in an OV-independent manner [337]. In addition, oncolytic viruses can affect the immunosuppressive tumor microenvironment (TME) by delivering immunostimulatory transgenes such as cytokines, T-cell costimulatory ligands, and checkpoint inhibitors [338,339,340].

Most RNA viruses have great potential as oncolytic virus platforms because of their temporary transgene expression, more steadfast replication, high immunogenicity, and tropism for tumor cells [336,341]. In addition, RNA viruses have the capacity to eliminate tumor cells faster than DNA viruses as they replicate in the cytoplasm without requiring access to the nuclei of target cells [342]. Oncolytic RNA viruses like reovirus, the measles virus, Newcastle disease virus, and Vesicular stomatitis virus (VSV) are present in ongoing clinical trials. At the same time, the vast majority of clinical trials of oncolytic vectors are conducted on the platform of DNA viruses such as adenovirus and herpes simplex virus (HSV) [343]. Although DNA viruses have relatively low immunogenicity compared to some RNA viruses, the presence of a larger and more stable genome makes them more attractive through simple genetic engineering (Table 2).

Despite the high therapeutic potential of tumor-selective OV mechanisms, it has been noted that a number of patients do not respond to OV therapy, in particular with monotherapy. A lack of efficacy and an unacceptable toxicity profile are the main reasons for the discontinuation of the use of certain viruses as platforms for OVs. Several factors may limit the effectiveness of OVs when used as systemic monotherapy.

First, as with gene delivery, a significant limitation for OV platforms is the presence of pre-existing immunity and, as a consequence, neutralizing antiviral antibodies. In the presence of pre-existing immunity, when administered systemically, the therapeutic effect of the agent will be reduced both due to binding by circulating neutralizing antibodies and due to the attack by virus-specific cytotoxic CD8+ T cells on host cells already infected with the virus [344]. Neutralizing antibodies primarily target viruses, such as adenovirus and HSV-1 that circulate in the human population or are used as vectors for vaccination. At the same time, the seroprevalence of vesicular stomatitis virus (VSV) and Newcastle disease virus (NDV) is much lower because their natural hosts are not humans. Pre-existing immunity typically does not restrict the efficacy of intratumoral OV therapy. Thus, in a model of immunocompetent hamsters, it was demonstrated that there was no significant influence of pre-existing immunity on the antitumor efficacy of the oncolytic adenoviral vector INGN 007, identical to wild-type human Ad5, upon intratumoral injection [345]. However, in the case of systemic delivery of oncolytics, neutralizing antibodies are the main reason for their reduced therapeutic efficacy in the immunized host. For example, one study reported that the absence of viral particles and transgene expression in tumors following systemic administration of the oncolytic virus VSV-GFP to preimmunized mice was caused by the presence of neutralizing antibodies, and not by cellular responses [346]. In addition, the presence of previously developed humoral immunity is the reason for the limited efficacy of systemically delivered treatments based on Ad5, HSV, and the measles virus in preclinical trials [65,347,348]. Systemic administration of OVs in most cases is preferable to local administration due to the possibility of treating metastases and hard-to-reach tumors [337]. Therefore, many modifications to OV therapy have been explored to avoid NAbs neutralization and improve therapeutic efficacy. Among these strategies are the use of non-human OVs, modification of epitopes, creation of a protective coating, and use of a cellular carrier system for delivery of OVs [346,349,350,351,352].

Secondly, the effectiveness of oncolytic virotherapy is negatively affected by the activation of antiviral immunity, including complement activation, cytokine synthesis, and macrophage activation, which contributes to the rapid elimination of OV [353]. Although genetic engineering modifications can improve tropism for tumor tissue and increase transgene expression, such engineering can lead to loss of virus infectivity and a decrease in its oncolytic activity [352].

In addition to the immune-related challenges associated with oncolytic viruses, there are also platform-specific nuances that may hinder their successful application. One example is the adenovirus vector. As mentioned previously, this vector was among the first used in gene therapy, is relatively well studied, and is currently manufactured at a large scale for vaccine production. It also possesses a relatively large cloning capacity. However, in addition to the widespread pre-existing immunity noted earlier, adenoviruses exhibit broad tissue tropism, enabling them to infect non-malignant cells. Furthermore, adenovirus enters cells through interaction with the coxsackie adenovirus receptor (CAR), which is generally expressed at low levels in cancer cells. Consequently, enhancing the efficacy of oncolytic virotherapy using adenovirus requires modifications to its fiber knob region to improve tropism for cancer cells [354].

Similarly, HSV-1 is another well-studied virus that has been approved for clinical application as T-VEC. At least 30 kb of its genome encodes non-essential genes, which can be replaced with therapeutic transgenes. However, as previously noted, widespread pre-existing immunity can lead to rapid clearance of the OV from circulation before it reaches its target [355]. Vaccinia virus is also extensively studied due to its use as a smallpox vaccine. It does not integrate into the human genome, thus eliminating the risk of insertional mutagenesis. Vaccinia has a cloning capacity exceeding 25 kb, replicates rapidly, can replicate under hypoxic conditions, and exhibits broad tropism for tumor cells. Notably, the vaccinia virus can infect tumor cells after intravenous injection, even in the presence of NAbs [356]. Despite these advantages, the vaccinia virus has demonstrated lower oncolytic efficiency compared to adenovirus [357]. Reovirus is another potential candidate for use as an oncolytic virus. It is known to be relatively non-pathogenic and has been shown to selectively kill tumor cells [358,359]. However, upon intravenous administration, reovirus is often neutralized, resulting in diminished oncolytic efficacy [358].

The measles virus is a pathogenic lymphotropic virus with a documented ability to induce tumor remission as early as 1971. Today, an attenuated vaccine strain has been developed that is deemed safe for clinical application. This strain can also be enhanced through genetic engineering to improve tropism and introduce therapeutic genes. Nevertheless, similar to many viral platforms, the measles virus suffers from premature viral clearance, and its clinical use is limited by the high seroprevalence of anti-measles antibodies in the general population [360].

Newcastle disease virus (NDV) is an avian virus characterized by species-selective antiviral immune response evasion mechanisms. In mammalian cells, NDV induces strong interferon responses and is capable of specifically replicating in malignant cells with disrupted antiviral immune response pathways. NDV also exhibits low seroprevalence in the human population. However, it can still elicit a robust neutralizing immune response that may prevent re-administration. Additionally, NDV has shown attenuated effects upon systemic injection due to immune-mediated clearance and poor tumor infiltration [361].

Vesicular stomatitis virus (VSV) is another promising candidate for oncolytic virotherapy. The prevalence of pre-existing immunity against VSV in the human population is low [362], and it carries a minimal risk of genomic integration [363]. VSV replicates rapidly, facilitating large-scale manufacturing [362]. Nonetheless, VSV has been shown to be neurotoxic and may undergo premature inactivation by the host immune system [362].

Challenges like immune response activation, immune-related side effects, viral tropism limitation, dosing approaches, and safety considerations are frequently encountered in both gene therapy viruses and oncolytic viruses. In addition, a unique and significant challenge to the effective use of oncolytic viruses is the physical barriers hampering the entry into solid tumors. The high cellular density with multiple cell types, both malignant and normal cells, and the abundant deposition of extracellular matrix in the tumor represent a major obstacle to entry of the circulating virus and spread within the tumor lesion. Rapid tumor growth in combination with a dense extracellular matrix, impaired blood supply, and lymphatic drainage contribute to the formation of increased interstitial fluid pressure. This hinders the effective diffusion of therapeutic drugs [364,365]. The use of enzymes that destroy the extracellular matrix is one of the most successful strategies to combat these barriers to tumor tissue. In order to achieve this goal, a variety of enzymes including trypsin, collagenase/dispase, matrix metalloprotease 9, MMP1/8, hyaluronidase, and decorin have been effectively utilized to enhance viral concentration in solid tumors through exogenous delivery or incorporation into the viral expression cassette [366,367,368,369].

Thus, the criteria for viral platforms to produce oncolytic vectors differ from those for viral vectors designed for gene therapy. This is primarily attributed to the qualities of the tumor, including its type, physical obstacles within the tumor, heterogeneity, and an immunosuppressive microenvironment [364]. For example, the size of the virus is important for the effectiveness of oncolytic virotherapy. Smaller viruses can more readily infiltrate dense extracellular matrix networks and disseminate within the tumor, whereas larger viruses are more effective in delivering therapeutic genes. In addition, the presence of a viral envelope is a crucial consideration in selecting a viral platform. Enveloped viruses have less effective oncolytic properties compared to naked viruses, and they are more prone to be cleared by the host immune system. In addition, tumor tropism, seroprevalence, immunogenicity, and transgene insertion capability are important factors to consider when selecting a viral platform for an oncolytic virus. (Table 2).

## 6. Conclusions

The use of viral vectors in gene therapy has revolutionized the treatment of various conditions and genetic disorders. However, despite some significant clinical success, huge challenges remain that limit their broader application. All the viral vectors analyzed in this review have pros and cons and their application to specific conditions can be affected by different issues including cost-effectiveness, production yield, toxicity, and the ability of the organism to neutralize their therapeutic properties. Despite the aforementioned disadvantages of viral vectors, researchers are focused on developing various strategies to overcome these challenges. Novel viral vector serotypes are being created that can evade pre-existing neutralizing antibodies, ensure precise tropism, and provide higher yields during production. Enhanced production cell lines, along with improved production, purification, and quality control methods, are being developed to increase vector yield, reduce costs, and enhance the safety of viral vector-based therapies. Immune suppression regimens are also being explored to prevent neutralizing antibody formation and reduce the risk of immune-related adverse events in patients. Simultaneously, other viruses are being investigated as potential candidates for safer and more efficient viral vector platforms. Nonetheless, further research is necessary to refine these approaches and ensure that viral vectors can be safely and effectively used across a wider range of therapeutic applications. Future developments in gene therapy will likely focus on improving vector efficiency, reducing side effects, and expanding the range of treatable conditions through innovative vector engineering and delivery techniques.

## Figures and Tables

**Table 1:** Characteristics of DNA-based and RNA-based viral vectors for gene therapy.

	DNA-Based Viral Vectors			RNA-Based Viral Vectors		
	Adenoviral	AAV	Baculoviral	Gamma-Retroviral	Lentiviral	Foamy Viral
**Virion size**		20–25 нм	30–60 nm × 250–300 nm	80–120 nm	80–120 nm	80–120 nm
**Packaging capacity**	8 kb (replication defective) 30 kb (helper-dependent)	<5 kb	>38 kb	<8 kb	<8 kb	<12 kb
**Vector genome forms**	Episomal	Episomal	Episomal	Integrated	Integrated	Integrated
**Gene expression duration**	Short	stable long-term expression in non-dividing or slowly-dividing cells (years)	Transient (usually no more than 14–21 days)	stable long-term (years) expression	stable long-term (years) expression	stable long-term (years) expression
**Tropism**	Broad	Dividing and non-dividing cells	Dividing and non-dividing cells	Dividing cell	Dividing and non-dividing cells	Dividing cells
**Preexisting immunity**	Yes	Yes	Low	Low	Low	Low
**Safety**	Inflammatory response, high toxicity	Immune response and immune-mediated toxicity	High safety (but more studies are needed to confirm the safety in in vivo and ex vivo applications)	High risks of insertional mutagenesis	Reduced risk of insertional mutagenesis	Reduced risk of insertional mutagenesis
**Advantages**	Efficient transduction of most tissues -non-integrating virus -high cloning capacity	-relatively low immunogenicity -non-integrating virus -high transduction efficiency in both dividing and non-dividing cells -the presence of tropism for certain tissues and cell types	-large cloning capacity -non-integrating virus -high transduction efficiency in both dividing and non-dividing cells -high safety, since the virus does not infect human cells.	-low immunogenicity -stable long-term expression	-low immunogenicity -stable long-term expression -high transduction efficiency in both dividing and non-dividing cells	-cloning capacity is the largest among retroviruses -reduced risk of insertional mutagenesis -low immunogenicity -stable long-term expression
**Limitations**	Initiates strong inflammatory response	-small cloning capacity -can elicit strong immune response and immune-mediated toxicity -pre-existing neutralizing antibodies to many serotypes -loss of episomes in replicating cells -presence of genomic integration -high cost -ineffective production strategies	-transient gene expression -inactivated by the complement system and causes acute activation of the innate immune response -fragility and low stability of the viral envelope -requires resources for insect cell cultivation	-insertional mutagenesis -formation of replication-competent viral particles -fragility and low stability of the viral envelope	-insertional mutagenesis -formation of replication competent viral particles -fragility and low stability of the viral envelope	-dependence of integration on cell division -pseudotyping technologies have not been developed -fragility and low stability of the viral envelope
**Clinical applications**	-	Luxturna, Zolgensma, Hemgenix, Elevidys, and Roctavian are approved for the treatment of hereditary diseases	-	Tecartus and Yescarta are approved for CAR-T tumor therapy	Kymriah, Abecma, Breyanzi, and Carvykti are approved for the treatment of malignant neoplasms; Libmeldy is approved for the treatment of metachromatic leukodystrophy	-

**Table 2:** Characteristics of viral platforms for oncolytic viruses.

	DNA-Based Oncolytic Viruses			RNA-Based Viral Oncolytic Viruses			
	Adenovirus	Herpes Simplex Virus	Vaccinia Virus	Reovirus	Measles Virus	Newcastle Disease Virus	Vesicular Stomatitis Virus
**Virion size**	90–100 nm	200 nm	270 × 350 nm	85 nm	100–300 nm	100–300 nm	185 × 75 nm
**Genome size**	26–45 kb	154 kb	190 kb	23 kb	16 kb	15 kb	11 kb
**Envelope**	Naked	Enveloped	Enveloped	Naked	Enveloped	Enveloped	Enveloped
**Seroprevalence**	+	+	+	+	+	-	-
**Transgene packaging possible**	+	+	+	+	+	+	+
**Advantages**	-the feasibility of manufacturing high viral titers -ease of genome manipulation -large cloning capacity	-large cloning capacity	-fast and efficient spreading of the virus due to high-speed and active life cycle -does not integrate into the host genome - large cloning capacity - well-studied genome	-tumor-specific without genetic modifications -has been shown to be effective as a monotherapy	- tumor-specific without genetic modifications -an excellent safety record	-tumor-specific without genetic modifications -does not integrate into the host genome -lack of pre-existing immunity in humans	-does not integrate into the host genome -has a fast kinetic cycle -lack of pre-existing immunity in humans
**Disadvantages**	-extensive tissue tropism -high levels of pre-existing immunity - hepatic adsorption	-high levels of pre-existing immunity -quickly eliminated by the immune system after systemic injection -can cause a cytokine storm in the body with a high viral load -hepatic adsorption	- low oncolytic efficacy -potential difficulties with systemic delivery	-small cloning capacity -quickly eliminated by the immune system after systemic injection (e.g., via complement system and neutralizing antibodies) -activation of innate immunity prevents the spread of reovirus	- high levels of pre-existing immunity - challenges in scalable purification for clinical use	-quickly eliminated by the immune system after systemic injection (e.g., via complement system and neutralizing antibodies) - low effectiveness when administered systemically	-small cloning capacity -neurotoxicity in laboratory animals and humans -quickly cleared by the immune system (e.g., via complement system and neutralizing antibodies)
**Clinical applications**	Oncorine (H101) in combination with chemotherapy as a treatment for patients with late-stage refractory nasopharyngeal cancer	Imlygic (T-VEC) for the treatment of unresectable metastatic melanoma and Delytact (G47∆) for the treatment of malignant glioma	-	-	-	-	-

## Data Availability

No data was generated in this manuscript.
